# Environmental (in)dependence of a hybrid zone: Insights from molecular markers and ecological niche modeling in a hybrid zone of *Origanum* (Lamiaceae) on the island of Crete

**DOI:** 10.1002/ece3.2560

**Published:** 2016-11-16

**Authors:** Michael Bariotakis, Konstantina Koutroumpa, Regina Karousou, Stergios A. Pirintsos

**Affiliations:** ^1^ Department of BiologyUniversity of CreteHeraklionGreece; ^2^ School of BiologyAristotle University of ThessalonikiThessalonikiGreece; ^3^ Botanical GardenUniversity of CreteRethymnonGreece; ^4^ Present address: Department of Systematic and Evolutionary BotanyUniversity of ZürichZürichSwitzerland

**Keywords:** contact zone, genotypic classes, hybridization, management practices, niche modeling, *Origanum*, spices

## Abstract

The role of environment and the relative significance of endogenous versus exogenous selection in shaping hybrid zones have been crucial issues in the studies of hybridization. Recent advances in ecological niche modeling (ENM) offer new methodological tools, especially in combination with the genotyping of individuals in the hybrid zone. Here, we study the hybrid zone between the widely known spices *Origanum onites* and *Origanum vulgare* ssp. *hirtum* in Crete. We analyze the genetic structure of both parental taxa and their hybrid *Origanum *×* intercendens* using AFLP markers on 15 sympatric and 12 allopatric populations and employ ecological niche modeling and niche similarity tests to study their niche patterns. We complement these analyses with seed viability measurements. Our study revealed that the hybridizing taxa *O. onites* and *O. vulgare* ssp. *hirtum* and the resulting genotypic classes showed geographical and environmental niche similarities based on the predictions of ENMs and the subsequent similarity tests. The occurrence of the hybrid zone is not directly dependent on environmental factors which favor the fitness of the hybrid compared to the parental taxa, but rather on aspects such as historical factors and management practices, which may contribute to the localization and maintenance of the contact zone between parental species. Our results suggest that if a minimum required niche differentiation between genotypic classes is not achieved, environmental dependence might not have a prominent role on the outcome of the hybridization.

## 1 Introduction

The phenomenon of hybridization has drawn the scientists’ interest for many decades, with a lot of ongoing debates concerning the role of hybridization in evolution. Hybridization, which seems to be quite common in plants (Ellstrand, Whitkus, & Rieseberg, 1996; Whitney, Ahern, Campbell, Albert, & King, 2010), is considered by some as evolutionary noise (e.g., Schemske, 2000), while the majority regard it as a phenomenon with an important evolutionary impact (e.g., Abbott et al., 2013; Arnold, 1997; Rieseberg et al., 2003). The significance of hybridization in evolution has been demonstrated among others in (a) hybrid‐mediated speciation events (via allopolyploidization, e.g., Perný, Tribsch, Stuessy, & Marhold, 2005; Ma, Li, Vogl, Ehrendorfer, & Guo, 2010 or homoploid hybrid speciation, e.g., Fjellheim, Holten Jørgensen, Kjos, & Borgen, 2009; Brennan, Barker, Hiscock, & Abbott, 2012), (b) genetic homogenization and the subsequent fusion of genetically distinct lineages via gene flow and recombination (Seehausen, 2004; Seehausen, Takimoto, Roy, & Jokela, 2008), and (c) the transfer of adaptive characters among species via introgression, providing species with the opportunity to occupy novel habitats or increase their fitness in their existing habitats (e.g., Campbell, Snow, & Sweeney, 2009; Whitney et al., 2010).

According to Barton and Hewitt (1985), the term hybrid itself can be misleading, since it does not refer to a single entity but rather a number of possible genotypic combinations, which may be categorized in distinct genotypic classes (Arnold, 1997). The identification and subsequent analysis of such classes can reveal variation that may correspond to divergence in ecological characteristics (Swenson, 2006) or fitness (Arnold & Hodges, 1995), also providing a more structured foundation for the comparison of hybrids to their parental taxa.

Hybrid zones provide a useful geographical context for studying the various outcomes of hybridization between genetically divergent populations that come in contact, cross‐mate, and produce individuals of admixed ancestry. The extent of genetic variation among participating individuals can be examined by identifying the variety of different genotypic classes occurring in a hybrid zone. Jiggins and Mallet (2000) used the graphical distribution of genotypic classes to characterize two contrasting types of hybrid zones. Unimodal hybrid zones describe cases where intermediate hybrid genotypes predominate, forming a “hybrid swarm”, whereas bimodal hybrid zones consist mainly of genotypes similar to those of the parental forms, with only few intermediate genotypes present. Those types of hybrid zones have been indicated to differ in the strength of prezygotic and postzygotic isolation mechanisms. In unimodality, those mechanisms are not sufficient to hinder the extensive formation of intermediate forms, whereas in bimodality, quite strong prezygotic isolation seems to occur but postzygotic isolation mechanisms are documented in fewer cases (Jiggins & Mallet, 2000). In natural hybrid zones, there are also cases of intermediate or “flat” genotypic distributions, representing zones transition between unimodal and bimodal distributions (Jiggins & Mallet, 2000). Knowing the pattern of genotypic structure in a hybrid zone can provide valuable insight into the ecological and evolutionary processes participating in the generation and further maintenance of a hybrid zone.

Classic theories have highlighted the relative significance of endogenous (environment independent) versus exogenous (environment dependent) selection affecting the structure and maintenance of hybrid zones (see Arnold, 1997; Barton, 2001; Barton & Hewitt, 1985). The environment‐independent model under the term “tension zone” assumes that endogenous selection against hybrids is acting in balance with the dispersal of the parental taxa in a hybrid zone (Barton & Hewitt, 1985), whereas the “bounded hybrid superiority” model assumes that hybrids are more fit in a certain environment than either of their parents, indicative of environmental dependence (Moore, 1977). Recently, incorporation of ecological niche models (ENMs) on analysis of hybrid zones has been proven useful in the characterization of those two prominent types of hybrid zones (Swenson, 2006, 2008). Here, we suggest further implementation of the ENMs in combination with both morphological and molecular markers within the (new) integrated framework for the hybrid zone models (Curry, 2015), which unifies the current models along a continuum of selection pressures.

Hybridization is a common phenomenon in the genus *Origanum* L. (Lamiaceae) and is regarded as the most important speciation mechanism for the genus (Ietswaart, 1980). Ietswaart (1980) supported this trend for speciation via hybridization primarily on intermediate morphological characters between representatives in the tribe of *Saturejeae* recognized in extant *Origanum* species. He hypothesized that in late Pliocene and Pleistocene, climatic changes that led to more arid conditions forced *Origanum* species to move toward the mountains where they came into contact with each other and related species from *Saturejeae*, hybridized and formed new species*. Origanum* consists of 44 species and has its center of diversity in the East Mediterranean (ca. 75% of the species) (Ietswaart, 1980). On the island of Crete (Greece), the genus *Origanum* is represented by five species and two hybrids (Karousou & Kokkini, 2003). In this study, we investigate the natural hybridization among *Origanum vulgare* L. ssp. *hirtum* (Link) Ietswaart and *O*riganum *onites* L., and their hybrid *Origanum* x *intercedens* Rech. f. on the island of Crete. *Origanum vulgare* ssp. *hirtum* mainly occurs in West and Central parts of Crete, whereas *O*. *onites* is mainly recorded in East and Central Crete (Karousou & Kokkini, 2003). The native distribution of *O. vulgare* ssp*. hirtum* expands into Albania, Croatia, Bulgaria, Greece, and Turkey, while that of *O. onites* in Greece, Sicily (Italy), and Turkey. *Origanum vulgare* ssp. *hirtum* and *O*. *onites*, known as “Greek” and “Turkish oregano,” respectively, are two of the most important commercial aromatic‐culinary herbs in the genus, with a significant role in the Mediterranean diet. In Crete, the consumption and the commercial use of the above two species are almost exclusively linked to the harvesting of natural populations. *Origanum* × *intercedens* is a natural hybrid that was first described by Rechinger (1961) from the island of Evoia and has since been found on the islands of Nisyros, Lesvos, Mykonos, Crete, and in a locality of SW Turkey (Ietswaart, 1980, 1982; Karousou, Hanlidou, & Kokkini, 2002; Kokkini & Vokou, 1993). In none of these cases was a hybrid zone reported, while in most cases, only one individual was found. The hybrid is distinguished from its parental taxa by inflorescence form and calyx shape. *Origanum* × *intercedens* has an intermediate inflorescence form between the paniculate of *O*. *vulgare* ssp. *hirtum* and the corymb of *O*. *onites*. Hybrid calyces are two‐lipped with three teeth on the upper lip and two on the lower lip, whereas *O*. *onites* has one‐lipped calyces and *O*. *vulgare* ssp. *hirtum* has actinomorphic calyces (Ietswaart, 1980; Rechinger, 1961). Previous studies on naturally occurring *O*. × *intercedens* have focused mainly on the essential oil composition of the hybrid and its parental taxa (Gounaris, Skoula, Fournaraki, Drakakaki, & Makris, 2002; Kokkini & Vokou, 1993; Skoula, Gotsiou, Naxakis, & Johnson, 1999), whereas information on its genetic relationship to the parental taxa is restricted to the use of RAPDs on 16 individuals collected from one population in Crete (Gounaris et al., 2002).

This study combines molecular data with ecological niche modeling and data of seed viability measurements aiming to a more comprehensive understanding of the patterns and structure of an *Origanum* hybrid zone in Crete. Specifically, we analyzed the genetic structure of the hybrid zone, distinguishing different genotypic classes. We also explored distribution patterns and niche similarities in both geographical and environmental space among (i) morphologically assigned hybrids and parental taxa and (ii) the distinct genotypic groups revealed from molecular analysis, thus assessing the role of their environmental dependence in shaping the hybrid zone.

## 2 Materials and methods

### 2.1 Sampling

A hybrid zone was identified for the first time in the north central part of Crete, where *O*. × *intercedens* grows in sympatry with its parental taxa. Fifteen sympatric populations of the hybrid with both or one of its parents were found in the hybrid zone, all of which were sampled, as well as six allopatric populations of *O*. *onites* in the eastern and six allopatric populations of *O*. *vulgare* ssp. *hirtum* in the western part of the island, outside of their sympatric distribution range (Table S1, Figure S1). A minimum distance of 850 m in between has been used as the criterion for the distinction of populations.

The hybrid identification took place both in the field and in the laboratory using voucher specimens, based on the descriptions given by Rechinger (1961) and Ietswaart (1980, 1982). Calyx shape, which is a key diagnostic character for *Origanum* species, was studied for all the individuals used in this study (Figures S2 and S3). With respect to hybrids, we examined in detail the characteristics related to the morphology of the two‐lipped calyces. A high variation in the shape and size of the calyx teeth was observed and a dichotomous key was prepared in order to distinguish the hybrids by their calyx morphology (Table S2). We identified eight different hybrid calyx types (Figure S3), with the most common type (Type A) to be present in about 50% of *O. × intercedens* (Figure S4).

While sampling, particular care was given to avoid re‐sampling of random selected individuals and collect only few stems from each plant in order to avoid irreversible damage of the individuals in the field. Fresh leaves from 221 individuals in total were collected and stored in silica gel, and 211 of them were successfully used in molecular analysis.

### 2.2 DNA extraction and AFLP genotyping

The *Origanum* leaves were ground in liquid nitrogen, and the leaf powder was stored at −80°C. DNA extraction was performed using the innuPREP Plant DNA kit (Analytik Jena AG, Jena, Germany), starting with 50 mg of leaf powder and following the manufacturer's instructions. An extra cleaning step with chloroform–isoamylalcohol (24:1) was included in order to remove the remaining secondary metabolites.

The technique of amplified fragment length polymorphisms (AFLP) was based on the protocol developed by Vos et al. (1995) with some modifications (Data S1). Twenty‐four primer combinations of *Eco*RI‐ANN and *Mse*I‐CNN were tested, and four of them (Table S3) were chosen to be applied in all the samples. The amplified fragments of the selective PCRs were separated using the capillary sequencer ABI PRISM 3130xl (Applied Biosystems, Foster City, CA, USA), compared with the GeneScan™‐500 Liz™ size standard (Applied Biosystems), and the results were subsequently analyzed for the fragments’ presence/absence using the software GeneMapper4 (SoftGenetics, State College, PA, USA). The reproducibility of the AFLP genotyping was calculated using 20 DNA‐extracted samples randomly chosen to be replicated for the whole AFLP technique. The error rate per locus was estimated based on Bonin et al. (2004) and 65 of 870 initially scored bands produced from the four primer combinations (Table S3) were excluded based on a threshold of 20% error rate. This dataset of 211 individuals and 805 polymorphic markers that was further used in all the molecular analyses has a mean error rate of 3.9%, which is lower than a maximum of 5% suggested by Bonin et al. (2004). This error rate also falls within the range of ≤2 and >4% proposed in a phylogeographic analysis by Zhang and Hare (2012) for the identification of population substructure patterns using structure (The Pritchard Lab, Stanford University, Stanford, CA, USA) (Pritchard, Stephens, & Donnelly, 2000).

### 2.3 Molecular data analysis

Species‐specific markers were identified comparing the marker's frequencies among the two parental taxa for their allopatric and sympatric populations. Specifically, two criteria were followed for the characterization of an AFLP marker as species‐specific: (i) the markers’ frequency difference among the two parental taxa should be at least 0.75 or the marker should be absent in one taxon and (ii) the frequency of the marker in the other taxon should be at least 0.80.

A Bayesian clustering method implemented in structure2.3.3 (Falush, Stephens, & Pritchard, 2007; Pritchard et al., 2000) was chosen in order to infer the genetic structure using the AFLP dataset (211 individuals and 805 markers). The input file for structure was produced in R2.15.0 (R Development Core Team, 2013) using the R function “Structure.E” of function collection AFLPdat (Ehrich, 2006). In the software, we chose the admixture model and the model of correlated allele frequencies assuming that the individuals may have a mixed ancestry, while the degree of admixture (alpha) was chosen to be inferred from the data. For each cluster *K* (*K *=* *1–8), we performed 10 replicates of 300,000 burn‐in period steps of MCMC followed by 700,000 further iterations. For the selection of the optimal number of clusters (*K*) to explain the dataset, the values of the mean log probability (*LnP*(*D*)) were used for the calculation of Δ*Κ*, as described by Evanno, Regnaut, and Goudet (2005). For the optimal number of *K*, the membership coefficients of each cluster were calculated for every individual, estimating their ancestry proportions.

Additionally, we analyzed the AFLP dataset using newhybrids (Anderson & Thompson, 2002) which is a Bayesian clustering algorithm that calculates the posterior probability (*p*) for each individual to be assigned into six genotype frequency classes (two pure classes: Pure 1 and Pure 2 for the two parental species and four hybrid classes: F1, F2 and BC 1, BC 2 for the backcrosses). The algorithm was run for a burn‐in period of 100,000 sweeps followed by another 500,000 sweeps, and three independent runs were performed to check for consistency of the results. The results of newhybrids, however, have been taken into consideration with caution because of a main limitation. The algorithm is very sensitive in the correct classification of individuals into genotype frequency classes when the presence of informative species‐specific markers is limited (Anderson, 2008), which is the case in our dataset (see Results). The problem becomes more prominent when distinguishing between hybrid classes, with the classification into backcross categories being the most difficult (Anderson, 2008). Therefore, the output from structure was chosen over newhybrids to be used for subsequent analyses and will be discussed in greater detail in this study.

Principal coordinates analysis (PCoA) was performed to reveal the pattern of genetic variation among the individuals of the three taxa. For this ordination method, we created a distance matrix based on the Jaccard similarity coefficient for the AFLP dataset using the package ade4 (Dray & Dufour, 2007) in R.

### 2.4 Seed viability

Seed viability was measured using tetrazolium test on mature and healthy seeds collected and stored at room temperature. Seeds of six *O*. *onites*, five *O*. *vulgare* ssp. *hirtum,* and ten *O*. × *intercedens* individuals were immersed in water for 24 hr. Afterward, the seeds were sown on filter paper in Petri dishes, cut vertically using a razor blade, and immersed on a 1% 2,3,5‐triphenyltetrazolium solution for 30 min. The living seed tissue produces a red color, while dead tissues do not stain. Only a limited number of seeds could be used in the viability test as the seeds were collected along with the fresh leaves’ sampling in summer during the flowering period when the plants were not fruitful.

### 2.5 Ecological Niche Modeling

Spatial modeling of the niche for the two parental taxa and the hybrid was carried out using maxent 3.3.3k (AT&T Labs and Princeton University, Princeton, NJ, USA) (Phillips, Anderson, & Schapire, 2006), a maximum entropy‐based machine learning program that estimates the probability distribution for a species’ occurrence, based on the environmental constraints and its current occurrence data. maxent was chosen for two main reasons. First, it has been shown to work better than other methods in the presence of small sample sizes (Elith et al., 2006; Hernandez, Graham, Master, & Albert, 2006). Second, as it works with presence data only, it estimates the potential distribution of a species, instead of focusing in its realized distribution, an aspect that better suits the aims in this particular study (Phillips et al., 2006).

For the modeling, an initial set of 43 variables, which consisted of topographic (based on a digital elevation model produced by NASA; downloaded from http://www.cgiar-csi.org), climatic (obtained from the Hellenic National Meteorological Service and the Water Resource Department of the Prefecture of Crete), soil (retrieved from Harmonized World Soil Data Base, http://webarchive.iiasa.ac.at/Research/LUC/External-World-soil-database), and land‐cover data (collected by MODerate‐resolution Imaging Spectroradiometer, MODIS; downloaded from http://glcf.umd.edu) was selected. The number of variables was then reduced using two criteria: pairwise variable correlations and the relative contribution of variables in an initial modeling of the species. After removing variables with high correlation and low explanatory contribution, a dataset consisting of 13 variables was obtained (Table S4). The highest pairwise variable correlation for this final matrix, as expressed by Pearson's correlation coefficient, was 0.67. All environmental variables were set at a common spatial resolution of 600 m. Manipulations of the data, as well as the creation of all figures presented in results, were done using R and packages sp (Bivand, Pebesma, & Gomez‐Rubio, 2013) and SDMtools (VanDerWal, Falconi, Januchowski, Shoo, & Storlie, 2012).

Two occurrence datasets were created based on the coordinates of all sampled populations for use in both ecological niche modeling and all subsequent analyses. In the first (M), the three morphologically distinct taxa, *O. onites*,* O. vulgare* ssp. *hirtum,* and *O*. × *intercedens,* were considered, while the second dataset (G) comprised the genotypic groups resulting from the genetic structure analysis. As not all forms strictly correspond to distinct taxa, the term “entities” will hereby be used for the different types modeled.

In order to assess the relative importance of environmental factors and parental occurrence on the hybrid's distribution, a secondary predictor variable was created, by multiplication of the predicted probabilities of occurrence of both *O*. *vulgare* ssp. *hirtum* and *O*. *onites*, as given by maxent. This new predictor, termed “parental occurrence,” increases where both parental species have high probability of occurrence (indicating highly probable co‐occurrence) and decreases for low probability values of either or both parental taxa. Consequently, a second model was run for *O*. × *intercedens*, which included the parental occurrence predictor along with the 13 environmental variables, and the relative contribution of predictor variables was recorded.

maxent automatically calculates the area under the receiver operating characteristic curve (AUC), which provides an evaluation of a model's ability to distinguish between presence and background data (Phillips et al., 2006). For the calculation of AUC, the data were randomly split using a 10‐fold cross‐validation routine in maxent. This was individually done for both M and G datasets. In the case of M dataset, external evaluation was also conducted using a separate set of occurrence records, collected independently. This additional evaluation was only implemented in M dataset, as in the case of G dataset, the genetic information of the sampled individuals was not a priori available.

### 2.6 Niche variation

In order to check for divergence in the niche of the modeled entities and assess whether these are a result of environmental heterogeneity or actual niche differentiation, we employed the niche similarity test developed by Warren, Glor, and Turelli (2008). As this test requires the specific definition of a background area for each entity to be included, we followed Martínez‐Cabrera, Schlichting, Silander, and Jones (2012) and Theodoridis, Randin, Broennimann, Patsiou, and Conti (2013), conventionally defining a 20‐km buffer zone around the occurrence points for each entity as its background. This was considered to be a suitable approach based on short‐distance dispersal of seeds of *Origanum* by natural processes (Thanos, Kadis, & Skarou, 1995), further supported by preliminary tests using 2‐, 5‐, and 10‐km buffer zones, which showed no statistical difference with those of 20 Km. For consistency purposes, this background was used in all tests of niche similarity, in both geographical space and environmental space (G‐ and E‐space). Briefly, geographical space refers to the species’ distribution as plotted on a map, while species occurring in environmental space refer to a conceptual space defined by the environmental variables to which the species responds. Both Schoener's D metric (Schoener, 1968) and modified Hellinger distance I (Warren et al., 2008) were used to quantify niche overlap and consequently test for similarity. The whole procedure was carried out in ENM tools (Warren, Glor, & Turelli, 2010).

Apart from G‐space, niche similarity between the modeled entities was also evaluated in E‐space. Niche similarity tests in E‐Space were performed following the principal component analysis (PCA)‐based method developed by Broennimann et al. (2012), once again employing Schoener's D and modified Hellinger distance I. Additionally, the entities’ distributions along each independent variable were compared using the nonparametric Kruskal–Wallis and Wilcoxon tests to check for significance (Theodoridis et al., 2013). This part of the analysis was carried out in R, additionally employing ade4 (Dray & Dufour, 2007) and adehabitat (Calenge, 2006).

## 3 Results

Fifteen populations of *O*. × *intercedens* growing together with *O*. *onites* and/or *O*. *vulgare* ssp. *hirtum* were identified in the north central part of Crete forming a broad hybrid zone; twelve of them are recorded for the first time, while the other three populations were already known from Karousou and Kokkini (2003). Another population of *O*. × *intercedens*, recorded in the past by Karousou and Kokkini (2003) far away from the hybrid zone toward the West, has not been confirmed in this study, as there were no hybrids.

### 3.1 AFLP genotyping

The AFLP technique successfully applied in 211 individuals and the overall reproducibility was estimated to be 96.1%, based on the mean error rate calculated for 805 polymorphic markers. Comparing the allopatric populations of *O*. *onites* and *O*. *vulgare* ssp. *hirtum* on the basis of species‐specific markers, according to the criteria mentioned in the materials and methods, 15 species‐specific markers were found; thirteen of them characterize *O*. *onites* and the remaining two characterize *O*. *vulgare* ssp. *hirtum* (Table S5A). Comparing the sympatric populations of the two parental taxa, six species‐specific markers were identified (Table S5B) and only five markers were found to be species‐specific comparing all the populations (sympatric and allopatric) of the two parental taxa (Table S6). These markers characterize *O*. *onites*, while no species‐specific marker was found for *O*. *vulgare* ssp. *hirtum* in the last two comparisons.

**Figure 1:**
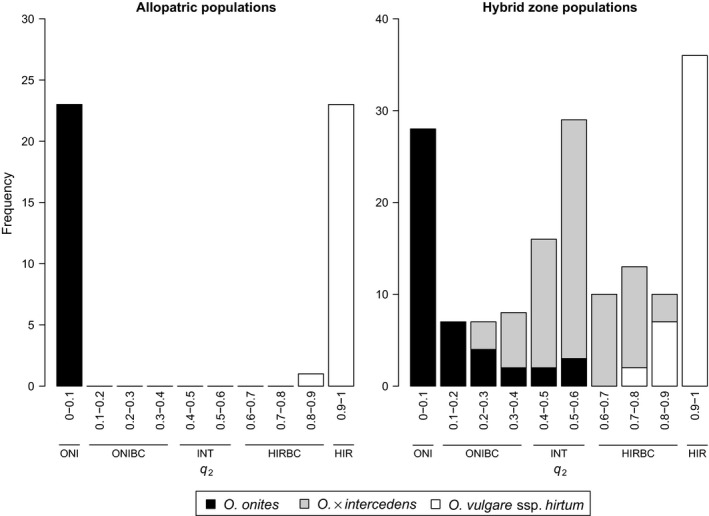
Histograms of *q*_2_ (estimated membership coefficient, from the genetic structure analysis for *K* = 2, corresponding to *O. vulgare* ssp. *hirtum*) in hybrid zone populations and allopatric populations. Low *q*_2_ values are indicative of *O. onites* and high *q*_2_ values of *O. vulgare* ssp. *hirtum*. Genotypic groups and their respective ranges of *q*_2_ are indicated below the ranges of the histogram classes: group ONI (*q*_2_ = 0–0.10), group ONIBC (*q*_2_ = 0.10–0.40), group INT (*q*_2_ = 0.40–0.60), group HIRBC (*q*_2_ = 0.60–0.90), and group HIR (*q*_2_ = 0.90–1)

In structure analysis, two clusters which clearly correspond to the two parental taxa (Figure S5) were revealed, as the highest Δ*Κ* value was observed for *K *=* *2 (Figure S6). Each individual was assigned in both clusters with two different estimated membership coefficients, *q*_1_ and *q*_2_ corresponding to *O*. *onites* and *O*. *vulgare* ssp. *hirtum,* respectively. For the hybrid zone, the membership coefficients of the two clusters were estimated for each of the 164 individuals (*O*. × *intercedens N* = 73, *O. onites N* = 46, *O. vulgare* ssp. *hirtum N* = 45) of the 15 populations. The genetic structure analysis revealed that 54.8% of the *O*. × *intercedens* individuals exhibited more or less equal degree of admixture for the two parental taxa (0.40 < *q*_2_ < 0.60) and were termed intermediate hybrids. Of the rest, 12.3% were genotypically closer to *O. onites* (0.10 < *q*_2_ < 0.40) and 32.9% closer to *O. vulgare* ssp. *hirtum* (0.6 ≤ *q*_2_ < 0.9); these individuals could most probably correspond to backcross hybrids and, in some cases, to advanced generation hybrids (Figure 1). Analysis of variance of the data showed no correlation between the hybrids’ calyx types and the genotypes (*p *=* *0.184). Most of the sympatric parental individuals of *O. vulgare* ssp. *hirtum* were pure (80%), with *q*_*2*_ **> 0.90, and only few individuals had a greater degree of admixture (0.7 < *q*_2_ < 0.9) corresponding to *O. vulgare* ssp. *hirtum*‐backcrosses (Figure 1). On the other hand, pure *O. onites* sympatric individuals (*q*_*2*_ **< 0.10) were less common (60.9%), while many samples of *O*. *onites* showed a broader range of admixture (0.1 < *q*_2_ < 0.6), including *O. onites*‐backcrosses, advanced generation, and intermediate hybrids (Figure 1). The allopatric *O. vulgare* ssp. *hirtum* (*N* = 24) and O*. onites* (*N* = 23) exhibited *q*_*2*_ **> 0.90 and *q*_*2*_ **< 0.10, respectively, values typical of pure taxa, with the only exception of an allopatric *O. vulgare* ssp. *hirtum* individual with *q*_2_ = 0.82 (Figure 1). The results of the genetic structure of allopatric individuals, on which we were primarily based in order to identify the range of *Q* values for pure taxa, were found to be in agreement with other studies (Cullingham, James, Cooke, & Coltman, 2012; Ortego, Gugger, Riordan, & Sork, 2014) concerning the use of a threshold of *q *=* *0.90 to distinguish between pure individuals (*q *≥* *0.90) and individuals of admixed ancestry (*q *<* *0.90). From the above analysis, five separate groups were identified based on the *q*_2_ values, which were further used as separate entities that comprise the G dataset of the ecological niche modeling: group ONI (*q*_2_ = 0–0.10), group ONIBC (*q*_2_ = 0.10–0.40), group INT (*q*_2_ = 0.40–0.60), group HIRBC (*q*_2_ = 0.60–0.90), and group HIR (*q*_2_ = 0.90–1) (Figure 1).

The results from newhybrids were broadly consistent with those from structure analysis. The distinction between pure and hybrid individuals was highly congruent between the two methods (Figure S5). All allopatric and sympatric parental individuals classified as pure in structure (*q *≥* *0.90) were also assigned to pure genotype classes in newhybrids (Figure S5). Admixed individuals were usually assigned to multiple hybrid classes with varying values of posterior probabilities (Figure S5). Most of the *O*. × *intercedens* individuals were assigned to F1 hybrid category (50.7%), while 20.6% were identified as F2 hybrids and the rest as backcrosses (26% *O. vulgare* ssp. *hirtum*‐backcrosses and 2.7% *O. onites*‐backcrosses). However, only 40% of the hybrid individuals were assigned to a single hybrid category with probability *p* > 0.85, which can be regarded as fairly high to confidently characterize the hybrid identity. In addition to that, the limitation of newhybrids algorithm to accurately classify hybrids into F1, F2, and backcrosses in the presence of few informative markers in the dataset (Anderson, 2008) hinders the unambiguous definition of hybrids.

**Figure 2:**
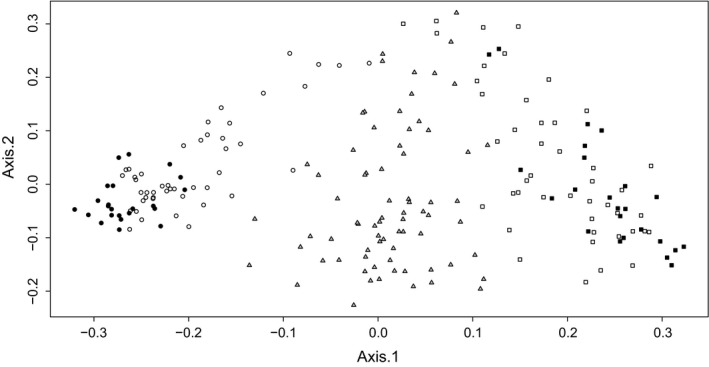
Principal coordinates analysis (PCoA) plot based on 805 AFLP markers for 211 individuals. The total explained variance in the two‐first axis was 17.4% (Axis.1 = 12.2%, Axis.2 = 5.2%). Circles represent *O. onites*, triangles represent *O. *× *intercedens,* and squares represent *O. vuglare* ssp. *hirtum*. For the parentals, black and white symbols correspond to allopatric and sympatric populations, respectively

The pattern of genetic variation presented in the ordination diagram of PCoA seems to follow the Bayesian clustering results. Along the first axis, the individuals of the two parental taxa were separated and the hybrids ordinated in between (Figure 2). Additionally, there is a direct correspondence between the position of each individual on the first axis of the PCoA diagram and the degree of admixture that each individual presents, based on structure.

### 3.2 Seed viability

The results of the tetrazolium test for seed viability are presented in detail in Table 1. Ninety‐four seeds from six *O. onites* individuals were examined, and 80.85% of them were viable with the percentages of seed viability per individual ranging from 72.2% to 93.3%. Viability for the 74 seeds of *O. vulgare* ssp. *hirtum* was lower (68.9%), and the percentages for each of the five samples varied from 46.15% to 83.3%. Even lower seed viability was recorded in the case of *O*. × *intercedens* (57%), where 107 seeds in total were examined and the percentages of the ten samples varied from 40% to 80%.

**Table 1:** Individuals used for the seed viability test, their population of origin, their genetic structure presented by the estimated membership of the cluster corresponding to *O. vulgare* ssp*. hirtum* (*q*_2_) in each individual's genotype, the percentage of seed viability, and the number of seeds used in parenthesis

Individual ID	Population	Genetic structure—*q*_2_	% seed viability (seeds)
*O. onites*			
107	ORI‐08	0.01	83.3 (12)
128	ORI‐15	0.6	72.2 (18)
129	ORI‐15	0.25	76 (25)
175	ORI‐16	0.01	93.3 (15)
183	ORI‐18	0.01	92.3 (13)
186	ORI‐19	0.03	72.7 (11)
			**80.85 (94)**
*O. vulgare* ssp*. hirtum*			
167	ORI‐14	0.99	83.3 (18)
169	ORI‐14	0.87	46.15 (13)
170	ORI‐15	0.96	66.6 (12)
199	ORI‐22	0.99	64.7 (17)
204	ORI‐23	0.91	78.6 (14)
			**68.9 (74)**
*O*. × *intercedens*			
2	ORI‐01	0.52	72.7 (11)
3	ORI‐01	0.8	40 (10)
21	ORI‐04	0.64	80 (10)
57	ORI‐11	0.48	50 (8)
59	ORI‐11	0.68	60 (6)
62	ORI‐12	0.77	41.6 (12)
73	ORI‐14	0.72	46.15 (13)
78	ORI‐15	0.61	61.1 (18)
X1	ORI‐02	–	50 (10)
X2	ORI‐04	–	77.8 (9)
			**57 (107)**

The mean percentage of seed viability and the total number of the examined seeds per taxon are in bold numbers. X1 and X2 correspond to individuals that were excluded from molecular analysis.

### 3.3 Ecological niche modeling

For both M and G datasets, cross‐validation of all models yielded AUC values above 0.8 (Table S7), which indicate good, while some values of the external evaluation were above 0.9, indicating excellent performance of the models. AUC values were also above 0.8 based on external evaluation (M dataset), with the exception of *O*. *onites*, which had an AUC score of 0.754 (Table S7).

**Figure 3:**
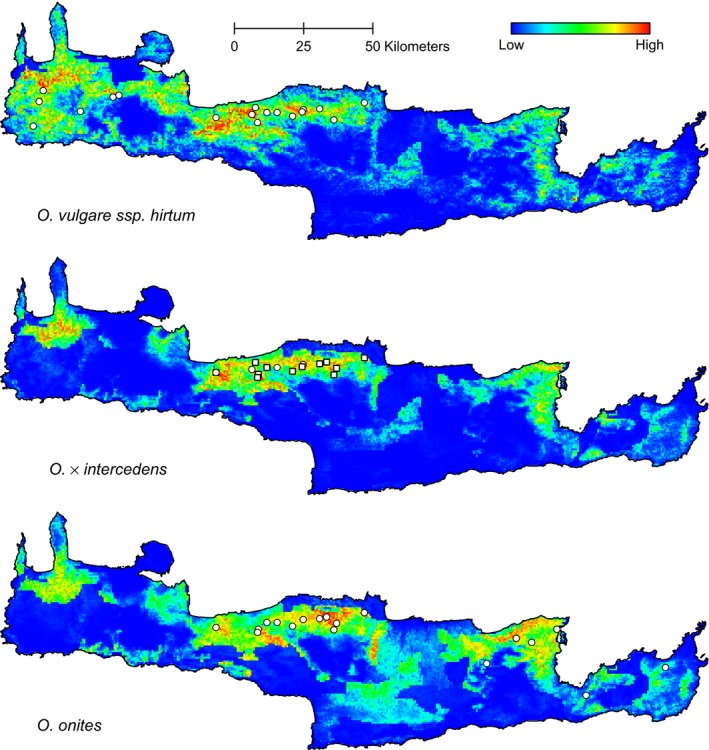
Predictions of the probability of occurrence for the M dataset. The white circles with black outlines represent the occurrence sites that were used as training points for the models. The white squares with black outlines correspond to newly found populations of *O. × intercedens*

Considering the predicted distribution of the M modeled entities (Figure 3), a striking pattern is apparent. Although the models accurately predict the areas where the three entities are expected to occur, their niches seem to extensively overlap. Apart from the center of the island, where the area of co‐occurrence and hybridization takes place, both parental taxa have at least one area of high probability (in the top 20th percentile of probability scores) in one another's distributions. Likewise, the hybrid's niche, although concentrated in the point of contact as expected, has localized extensions in both of its parental taxa areas of distribution.

**Figure 4:**
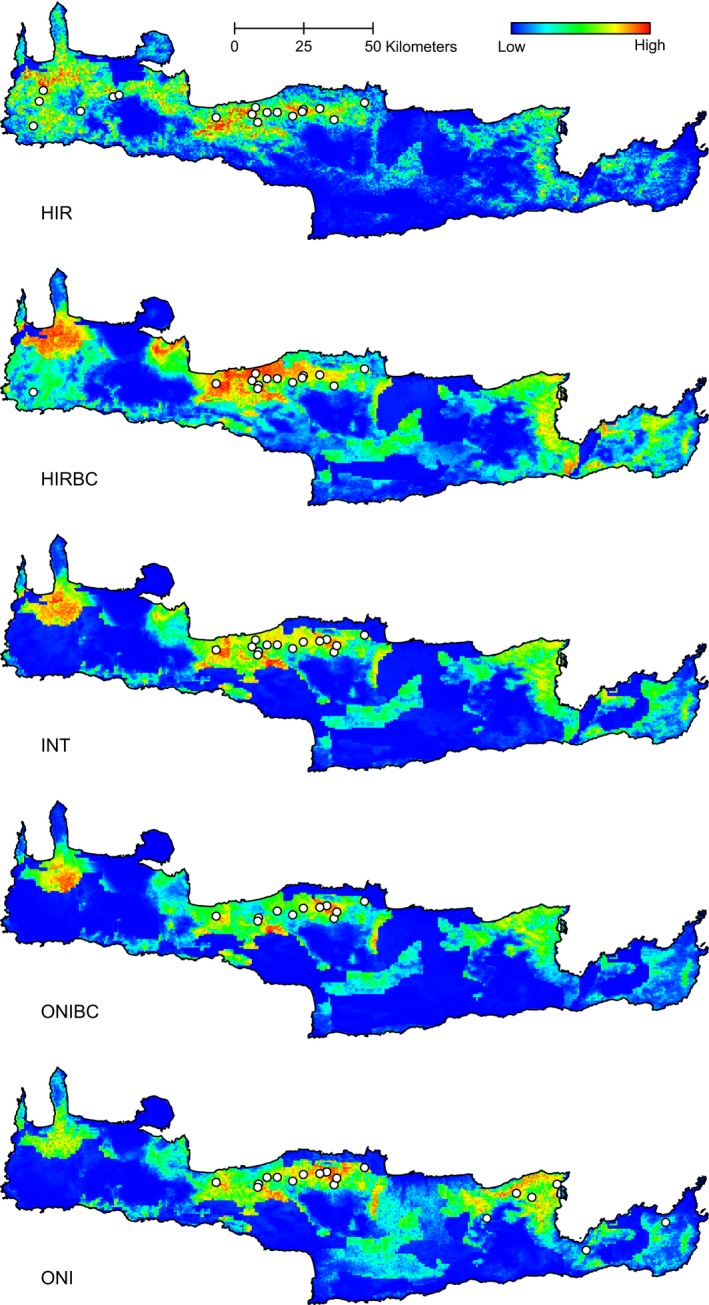
Predictions of the probability of occurrence for the G dataset. The white circles with black outlines represent the occurrence sites that were used as training points for the models. Genotypic groups correspond to the following ranges of *q*_2_: ONI 0–0.1, ONIBC 0.1–0.4, INT 0.4–0.6, HIRBC 0.6–0.9, HIR 0.9–1

The geographical similarities become more prevalent in the case of G dataset's modeled entities (Figure 4), where there is a smooth transition from one entity to the next, creating a gradient of probability distribution patterns from the extreme of *O*. *onites* (group ONI) to that of *O. vulgare* ssp. *hirtum* (group HIR).

Lastly, another striking aspect of the modeling results is the predominant importance of parental occurrence in comparison with all other explanatory variables, in the case of the mixed model of *O*. × *intercedens* (M dataset). The variable corresponding to co‐occurrence demonstrated a percent contribution of 96.2 and a permutation importance of 95.4, with the next most important variable, minimum temperature, demonstrating values of 1.1 and 2.1, respectively (Table S8).

### 3.4 Niche variation

Results concerning niche similarity tests in G‐space were consistent for both metrics across all pairs of entities for both the M and G datasets (Figures S7 and S8). Specifically, almost all pairs of entities were found to be significantly more similar than expected by the null distributions of their respective backgrounds, with three exceptions for Hellinger's I: *O. vulgare* ssp. *hirtum* in the background of *O*. × *intercedens* (M dataset), which was marginally nonsignificant (*p*‐value = 0.059) and HIR in the background of ONI and ONIBC (G dataset) which was found nonsignificant. Consequently, the produced pattern of this process confirms the similarities observed in the maps of predicted probability for the modeled entities.

The PCA‐based method for testing the niche similarity in E‐space resulted in patterns analogous to those of G‐space, with observed values of metrics D and I always situated toward (or beyond) the high end of the distributions of simulated values, indicating increased similarity of the tested pairs (Figures S7 and S9). However, the intensity of the pattern was lower in E‐space, as four of 12 tests performed in M dataset and 16 of 40 performed in G dataset were nonsignificant, according to the respective *p*‐values. Although this proportion of nonsignificant results may correspond to cases where the test has insufficient power to make inferences based on the environmental heterogeneity of the study area and the number of samples (Warren et al., 2008), the fact that the observed niche overlap always lies in the high end of simulated values strongly indicates a general tendency toward similarity rather than divergence.

Results of the Kruskal–Wallis and Wilcoxon tests on individual variables further support the above pattern of similarity, as only six of the 169 pairwise tests resulted in significant differences (Table S9). In both datasets, the differences were concentrated in the variables of maximum temperature and subsoil calcium carbonate. In particular, in the case of M dataset, the pair *O. vulgare* ssp. *hirtum* and *O. onites* was found to differ significantly for both these variables, while in the case of the G dataset, the pair HIR‐ONIBC differs in subsoil calcium carbonate, the pair HIRBC‐ONI differs in maximum temperature, and the pair HIR‐ONI differs in both.

## 4 Discussion

Until recently, natural hybrids between *O*. *onites* and *O*. *vulgare* ssp. *hirtum* on the island of Crete were considered as sporadic, because they were recorded from only four populations in areas where the two parental taxa co‐occur (Karousou & Kokkini, 2003). The present study extends the previously known distributional range of *O*. × *intercedens*, which is now known to consist of 15 populations forming a broad hybrid zone in the north central part of Crete in the area where the distributions of the two parental taxa overlap.

This hybrid zone was initially analyzed with respect to the genetic structure of populations using the AFLP technique which provides a variety of polymorphic markers derived from total DNA (Vos et al., 1995) and has been proven useful in the characterization of parental and hybrid genotypes (e.g., Eidesen, Alsos, & Brochmann, 2015; Galbany‐Casals, Carnicero‐Campmany, Blanco‐Moreno, & Smissen, 2012; Georgescu, Stefanakis, Kokkini, Katerinopoulos, & Pirintsos, 2016; Thompson, Anwarali Khan, Stangl, Baker, & Bradley, 2013; Zeng, Liao, Petit, & Zhang, 2011). The analysis of the polymorphic dominant markers revealed only five markers exhibiting substantial frequency differences among the parental taxa for both allopatric and sympatric populations. The identification of few species‐specific markers has been documented in other studies of interspecific hybridization, where AFLP markers have been used (Minder Rothenbuhler, & Widmer, 2007; Thompson et al., 2013; Wu & Campbell, 2005) indicating weak genetic differentiation across the genome in species under hybridization. Nevertheless, those species‐specific markers may be important for the maintenance even of a minimum degree of genetic differentiation preserving species integrity. The species‐specific markers we identified for the sympatric populations (six markers) were fewer than those for the allopatric populations (15 markers) of the parental taxa, indicating that gene flow in the hybrid zone may have also contributed to the reduction of genetic differentiation among the two parental taxa. Studies exploring the genetic diversity among individuals of *O*. *onites* collected from Mediterranean and Aegean regions of Turkey (Ayanoglu, Ergul, & Arslan, 2006) and individuals of *O*. *vulgare* ssp. *hirtum* collected from different sites in Greece (Katsiotis, Nikoloudakis, Linos, Drossou, & Constantinidis, 2009) show high intraspecific genetic diversity for each of the two parental taxa. This has been attributed primarily to their mating system that promotes cross‐pollination (Ayanoglu et al., 2006; Katsiotis et al., 2009). In general, there are no prezygotic barriers reported to hinder cross‐pollination in the genus (Ietswaart, 1980; Kitiki et al., 1997) and all the species possess the same chromosome number (2n = 30) (Bothmer, 1970). Those factors may facilitate crossabilities and gene flow among *Origanum* taxa growing in close proximity (Ietswaart, 1980).

The genetic structure analysis of the *Origanum* hybrid zone revealed a complex pattern, combining a bimodal and a unimodal distribution of genotypes corresponding to the morphologically identified parental taxa and the hybrid, respectively. Generally, we can observe a codominance of pure *O*. *onites* (*q*_*2*_ **< 0.10), pure *O*. *vulgare* ssp. *hirtum* (*q*_*2*_ **> 0.90), and intermediate hybrids (0.40 < *q*_2_ < 0.60). This genotypic distribution pattern departs from that of a “flat” hybrid zone that assumes an even mixture of all the different genotypic classes (Jiggins & Mallet, 2000). Our system shows an intermediate pattern that reflects one state in the continuum from unimodal to bimodal genotypic distributions in hybrid zones reported by Jiggins and Mallet (2000).

In many studies, morphology has been proven inadequate to characterize hybrids compared to molecular data analysis (e.g., Hardig, Brunsfeld, Fritz, Morgan, & Orians, 2000; Minder et al., 2007). In this study, the morphology of *O*. × *intercedens* was correctly associated with individuals of mixed ancestry. Still, there were some individuals morphologically assigned to *O*. *onites* exhibiting intermediate genotypes, together with genetically introgressed individuals of both *O*. *onites* and *O*. *vulgare* ssp. *hirtum*. In the latter cases, the phenotype did not mirror the genotypic composition and parental phenotypes are conserved.

The presence of a “hybrid swarm” with numerous intermediate genotypes in the *Origanum* hybrid zone could indicate the lack of strong pre‐ and/or postzygotic isolation barriers. In order to examine the potential lack of prezygotic isolation barriers, extensive studies including pollination/mating system, floral phenotypes, flowering time, pollen competition, and pollen–style incompatibilities must be carried out. *Origanum onites* and *O*. *vulgare* ssp. *hirtum* may differ in floral phenotypes concerning the calyx, the filaments, and the corolla shape but they demonstrate overlapping flowering times, while no pollen–style incompatibilities have been reported (Ietswaart, 1980). Despite the absence of detailed research concerning the various prezygotic isolation mechanisms acting among the two parental taxa, it seems that those mechanisms are not strong enough to prevent the formation of intermediate genotypes recorded in the hybrid zone.

With regard to postzygotic isolation, indications of weak barriers come from the genetic structure analysis of the *Origanum* hybrid zone and the seed viability test. Genetic structure analysis revealed the presence of various hybrid classes including intermediate hybrids (the great majority, 75% of them, identified as F1 and 25% as F2 hybrids, according to newhybrids) as well as advanced generation hybrids and backcrosses, implying an ability of hybrids to reproduce in nature. Additionally, individuals of *O*. × *intercedens* were found to possess viable seeds in proportions ranging from 40% to 80% indicating that at least some of these seeds could be able to germinate in nature. Nevertheless, it should be noted that the extent of postzygotic barriers and their effect on the structure of the hybrid zone cannot be based solely on seed viability, as over time small differences might accumulate and result in larger differences. Individuals with different degrees of admixture constantly obtained at least some viable seeds, while no clear association was observed between the percentage of seed viability and the admixture proportions. Although further research is necessary in order to draw clear conclusions on the relative importance on pre‐and postzygotic isolation barriers, strong reproductive isolation mechanisms acting in the hybrid zone seem unlikely on account of the observed genotypic pattern of “hybrid swarm”.

On the other hand, the strong bimodal distribution of parental genotypes in both allopatry and sympatry along with the considerable genetic divergence observed for the two parental taxa in PCoA ordination plot suggests that *O*. *onites* and *O*. *vulgare* ssp. *hirtum* remain genetically differentiated despite the extensive interspecific hybridization. Nevertheless, evidence of gene flow and substantial introgression exists in the hybrid zone, because of the identification of many *O. onites* and *O. vulgare* ssp. *hirtum*‐backcrosses. Although we have not studied in detail the nature of species boundaries in the case of *Origanum*, the fact that in sympatry, the parentals remain genetically differentiated despite the extensive introgression could be in line with the general idea of the semipermeable nature of species boundaries given by Wu (2001) and later by Harrison and Larson (2014). The differentiation of hybridizing species can be maintained despite the gene flow, due to varying permeability of particular genome region and therefore the hybridizing taxa often remain distinct for only a part of their genome (Harrison & Larson, 2014). Yet, a very important factor in the future of species integrity is the age of the contact zone between the two species. If the *Origanum* hybrid zone is recent, then the possibility that the species would merge or one would go extinct cannot be ruled out.

Over the last years, various GIS‐based methods using the distribution profiles of the parental and hybrid taxa succeeded in identifying hybrid zones where environmental selection acts upon their observed spatial patterns (Cullingham et al., 2012; Ortego et al., 2014; Wu, Ding, Yu, & Xu, 2015). Additionally, Swenson (2006, 2008) pointed out the utility of ENMs in testing classic theories concerning environment‐dependent (bounded hybrid superiority) or environment‐independent types (tension zone) of hybrid zones, and up to now, this type of modeling has been applied in various hybrid zone systems (e.g., Australian crickets, Kohlmann, Nix, & Shaw, 1988; North American avian, Cicero, 2004; Swenson, 2006; passerines in Europe, Engler, Rödder, Elle, Hochkirch, & Secondi, 2013). According to the bounded hybrid superiority model, the predicted distributions of the parental taxa could expand into the hybrid zone, while that of the hybrid should be restricted inside the hybrid zone boundaries (Swenson, 2006, 2008). In the case of the tension zone model, one or both the parental taxa and the hybrids should have predicted distributions that do not closely match but rather extend beyond their observed areas of occurrence (Swenson, 2006, 2008). Our results revealed a pattern where the predicted distributions of both parentals extend beyond the boundaries of the hybrid zone and beyond their observed distributions toward areas of one another's distributions. A similar pattern is also observed for *O*. × *intercedens*, where apart from the hybrid zone, there is high probability of occurrence in areas of distribution of both the parental taxa. These results seem to be in favor of the tension zone model.

Modeling of the G dataset, which is in agreement to both the idea of hybrids being a heterogeneous group of individuals with different genotypes (Arnold, 1997; Barton & Hewitt, 1985) and the suggestion to handle these genotypes as separate entities to investigate the extent of niche overlap among them (Swenson, 2006), gave even more noteworthy results toward the similarity of geographical patterns. Niche similarity tests not only confirmed the above mentioned patterns, but also allowed to further assess the structure of similarity between the studied morphological and genotypic groups. One noticeable outcome was that apart from the niche similarity demonstrated among separate hybrid groups and between hybrids and parental taxa, the parentals were also found to be significantly similar. These similarities were not restricted in geographical space, but were evident also in the environmental space, indicating that the environment might indeed not play the major role in differentiating the different entities. Furthermore, parental occurrence was found to have a dramatically higher contribution in predicting the distribution of *O*. × *intercedens* compared to the other environmental factors used, which additionally supports a tension zone model, where migration of parental taxa into the hybrid zone is one of the factors affecting its extent and localization (Barton & Hewitt, 1985). This is not to say that the distributions of the modeled entities themselves were independent of the studied environmental factors, as the ENMs had a very high predictive accuracy as shown by the AUC values. Instead, it is the lack of significant niche differentiation among the entities that leads to the environmental independence of the hybrid zone. Thus, both the tension zone and the bounded hybrid superiority models can be taken into consideration in view of the ENM predictions.

Despite that some ENM results appear to support a tension model, our system seems to be more complex. Although we have no experimental data on fitness of hybrids and parentals, it is expected that hybrids of advanced generation should be much less in a tension zone, and consequently, the hybrid zone should mainly consist of first‐generation hybrids (Gay, Crochet, Bell, & Lenormand, 2008; Jiggins & Mallet, 2000). The genetic structure of the *Origanum* hybrid zone cannot confirm this pattern, as there are a lot of individuals apparently corresponding to advanced generation hybrids and backcrosses based on both structure and newhybrids results. Moreover, other factors may implicitly play a role in the extent and localization of the hybrid zone, suggesting that an indirect role of environment cannot be excluded (Barton & Hewitt, 1985). The establishment of *O*. × *intercedens* and its observed genetic structure could be partially attributed to the traditional collecting protocols followed by locals for the Oregano collections during summer in Crete. Specifically, harvesting during the flowering period is limited only in part of the erect plant stems, while harvesting during fruiting periods involves in situ artificially induced dispersal of seeds, thus conserving local populations (WHO 2003). Additionally, the localization of the hybrid zone could also be in part attributed to the seed dispersal aided by human mobility along the main North Road Axis of Crete (NRAC) which crosses the entire northern part of the island from the western edge to the eastern one. There is no analogous road connecting the two edges of the southern part of Crete and only minor vertical to the NRAC roads service the driving to the south. An analogous transportation network node of human mobility in the hybrid zone could also be confirmed in historical times, such as in Roman era by Tabula Peutingeriana, which is the most representative piece of cartography of the Roman era, dated in 4th c. A.C. (335‐66) (see Pazarli, Livieratos, & Boutoura, 2007).

Overall, our study revealed that the hybridizing taxa *O. onites* and *O. vulgare* ssp. *hirtum* and the resulting genotypic classes showed geographical and environmental niche similarities based on the predictions of ENMs and the subsequent similarity tests. The occurrence of the hybrid zone is not directly dependent on environmental factors which favor the fitness of the hybrid compared to the parental taxa, but rather on aspects such as historical factors and management practices, which may contribute to the localization and maintenance of the contact zone between parental species. Subsequently, our results suggest that if a minimum required niche differentiation between genotypic classes is not achieved, environmental dependence might not have a prominent role on the outcome of the hybridization. Nevertheless, further analysis on the fitness of hybrids and parentals is needed, while the incorporation of environmental data in finer resolution is expected to improve the output of niche modeling. Hypothesis testing can be expanded in other *Origanum* species, and studies concerning species boundaries in the genus can be also implemented in the future.
