# Supplementary material for: Environmental (in)dependence of a hybrid zone: Insights from molecular markers and ecological niche modeling in a hybrid zone of Origanum (Lamiaceae) on the island of Crete
Source: Ecol Evol. 2016 Nov 16;6(24):8727–39. doi: 10.1002/ece3.2560 (PMC5192822; doi:10.1002/ece3.2560)
Supplement: Supplementary file 1 [file ECE3-6-8727-s001.pdf]

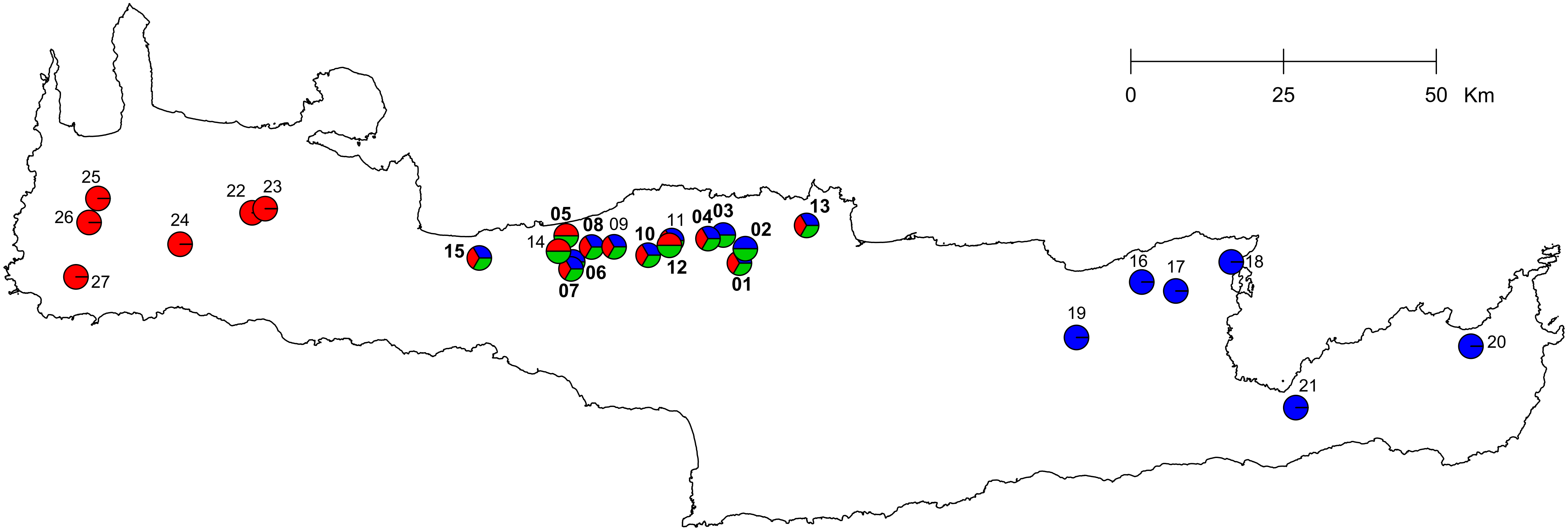

Fig. S1 Locations of sampling sites on the study area. Numbers indicate the number of the corresponding population (e.g. 01 refers to ORI-01). Numbers in bold correspond to new populations of *O.* × *intercedens*. Colors indicate the presence of each of the studied taxa, as follows: red - *O. vulgare* ssp. *hirtum*, green - *O.* × *intercedens*, blue - *O. onites*. Color portions on the pies in the central area represent the proportion of individuals per sampling site in the hybrid zone.

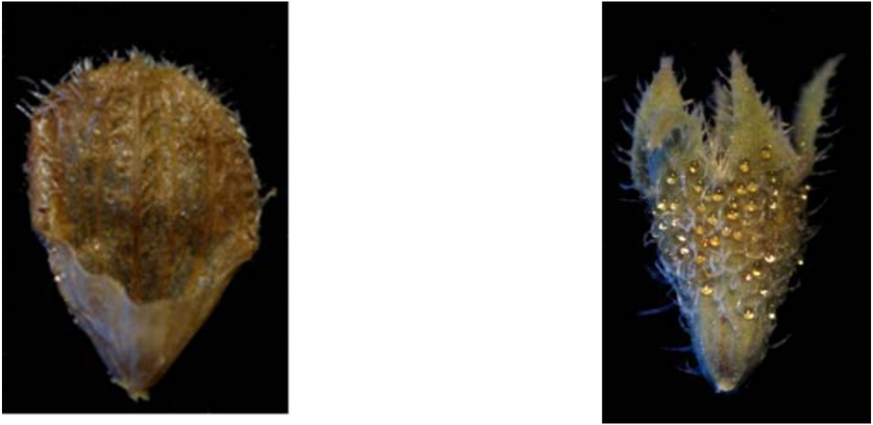

A B

Fig. S2. Calyx of *O. onites* (A) and *O. vulgare* ssp. *hirtum* (B)
