# Supplementary material for: Environmental (in)dependence of a hybrid zone: Insights from molecular markers and ecological niche modeling in a hybrid zone of Origanum (Lamiaceae) on the island of Crete
Source: Ecol Evol. 2016 Nov 16;6(24):8727–39. doi: 10.1002/ece3.2560 (PMC5192822; doi:10.1002/ece3.2560)
Supplement: Supplementary file 2 [file ECE3-6-8727-s002.pdf]

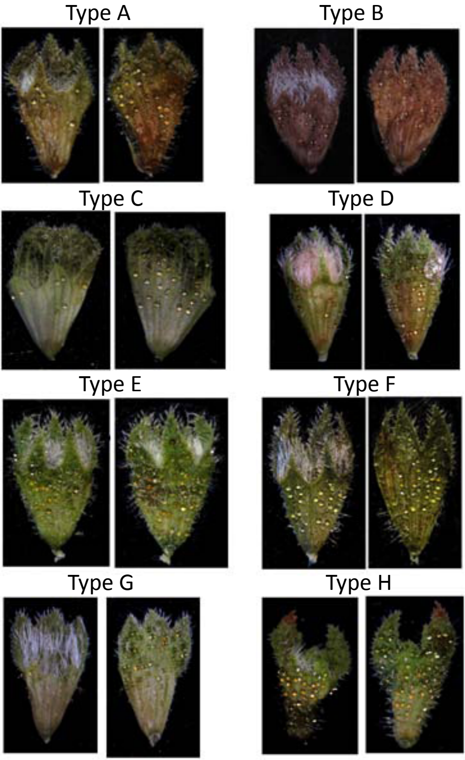

Fig. S3 Photographs of *Origanum* × *intercedens* calyx types A-H. For each pair, the image on the left corresponds to the lower lip, while the image on the right corresponds to the upper lip.
