# Supplementary material for: Environmental (in)dependence of a hybrid zone: Insights from molecular markers and ecological niche modeling in a hybrid zone of Origanum (Lamiaceae) on the island of Crete
Source: Ecol Evol. 2016 Nov 16;6(24):8727–39. doi: 10.1002/ece3.2560 (PMC5192822; doi:10.1002/ece3.2560)
Supplement: Supplementary file 3 [file ECE3-6-8727-s003.pdf]

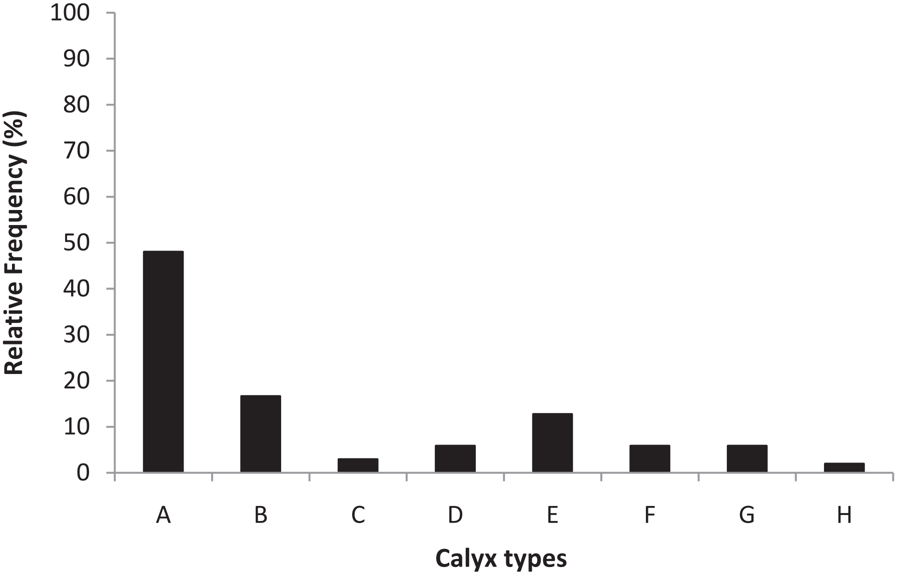

Fig. S4 Relative frequency of the *O.* × *intercedens* calyx types for the sampled individuals.

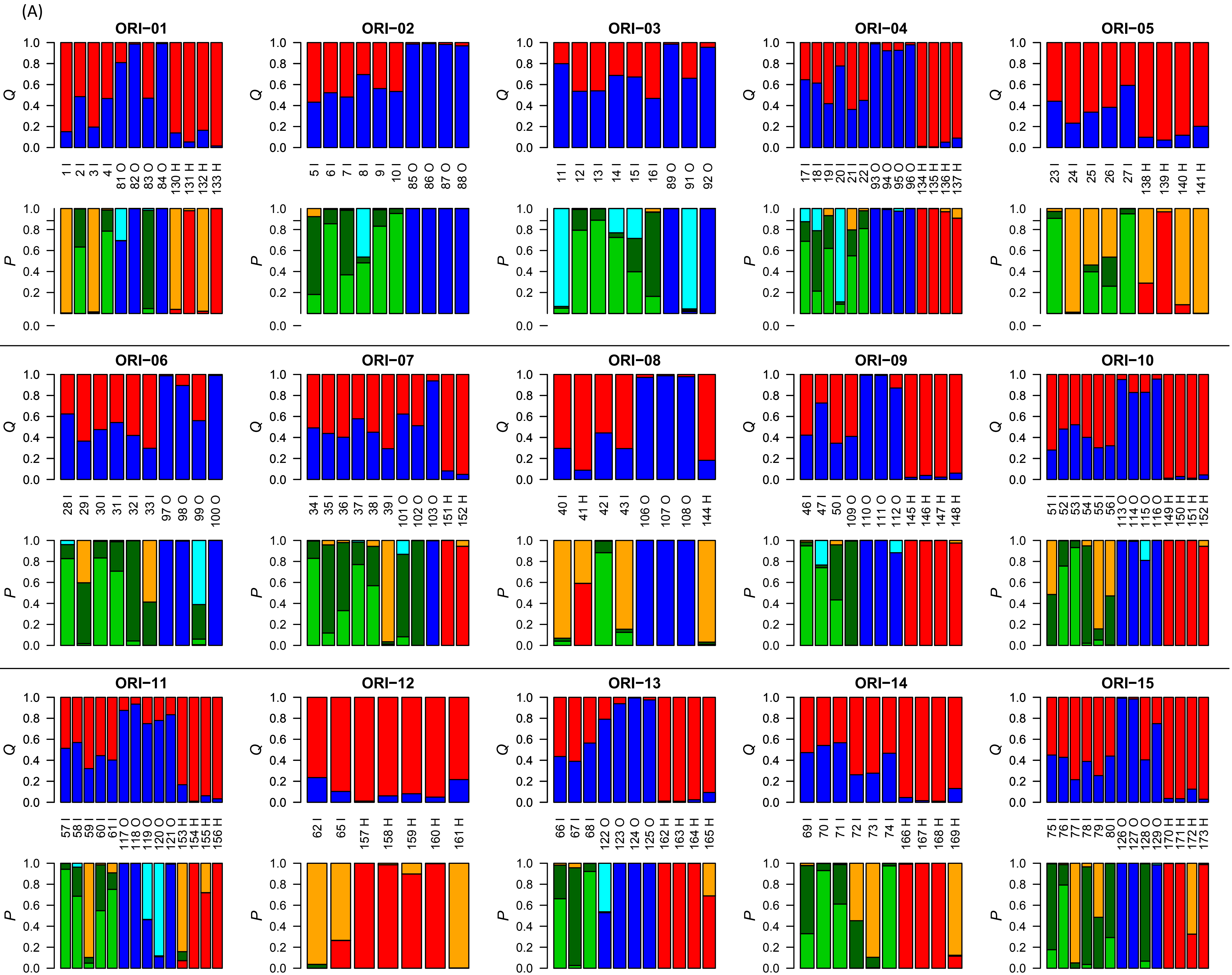

Fig. S5 Bar plots of *Q*, the estimated membership coefficients for each individual in each cluster from STRUCTURE, and *P*, the posterior probability estimated from NEWHYBRIDS. The colors correspond to membership values for each genetic category, as shown in the legend. Panel A shows sympatric populations, while panel B shows allopatric populations. Below the bars are the individuals' numbers, along with a letter that indicates the taxa (O for *O. onites*, I for *O.* × *intercedens* and H for *O. vulgare* ssp. *hirtum*).

(B)

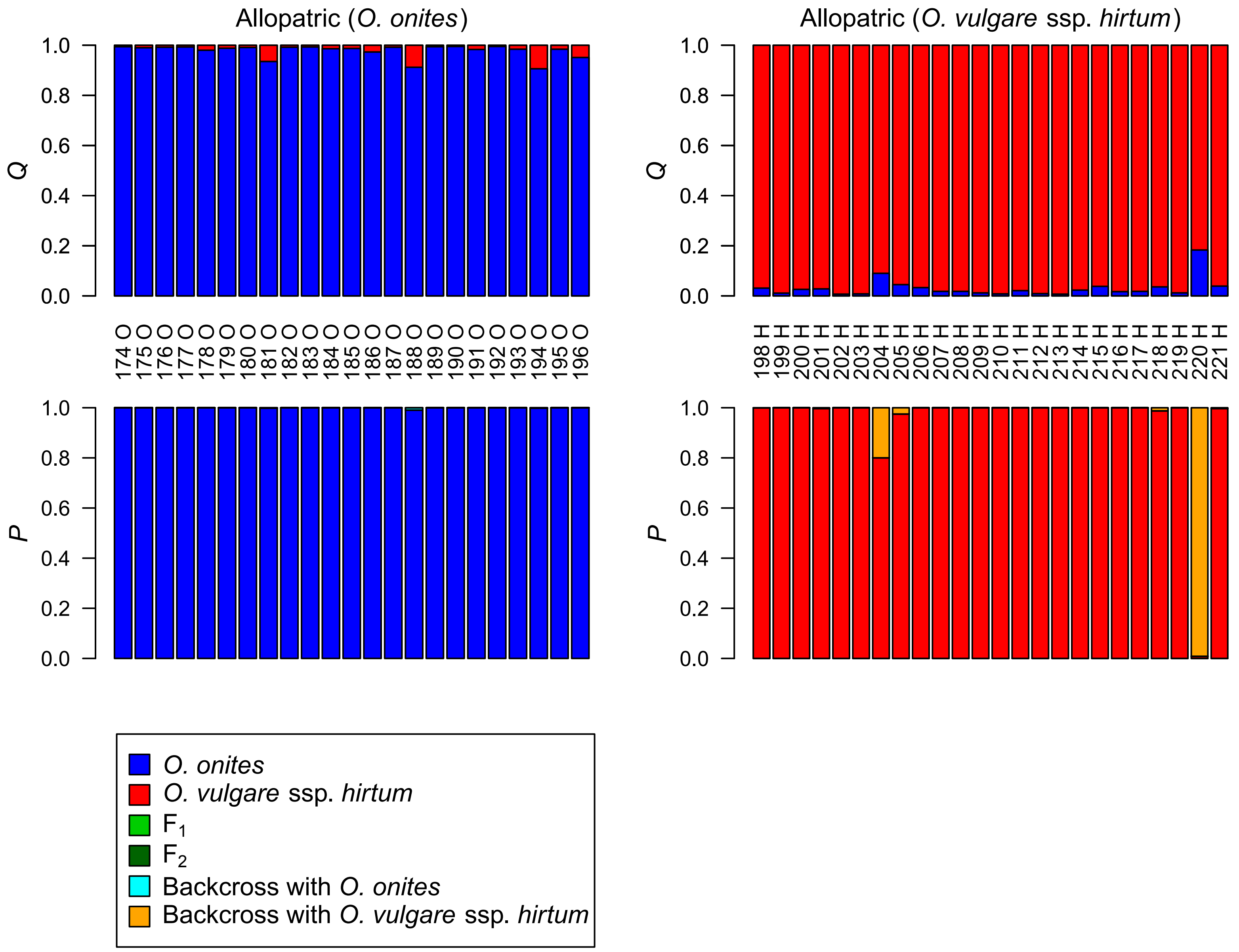

Fig. S5 (Continued)

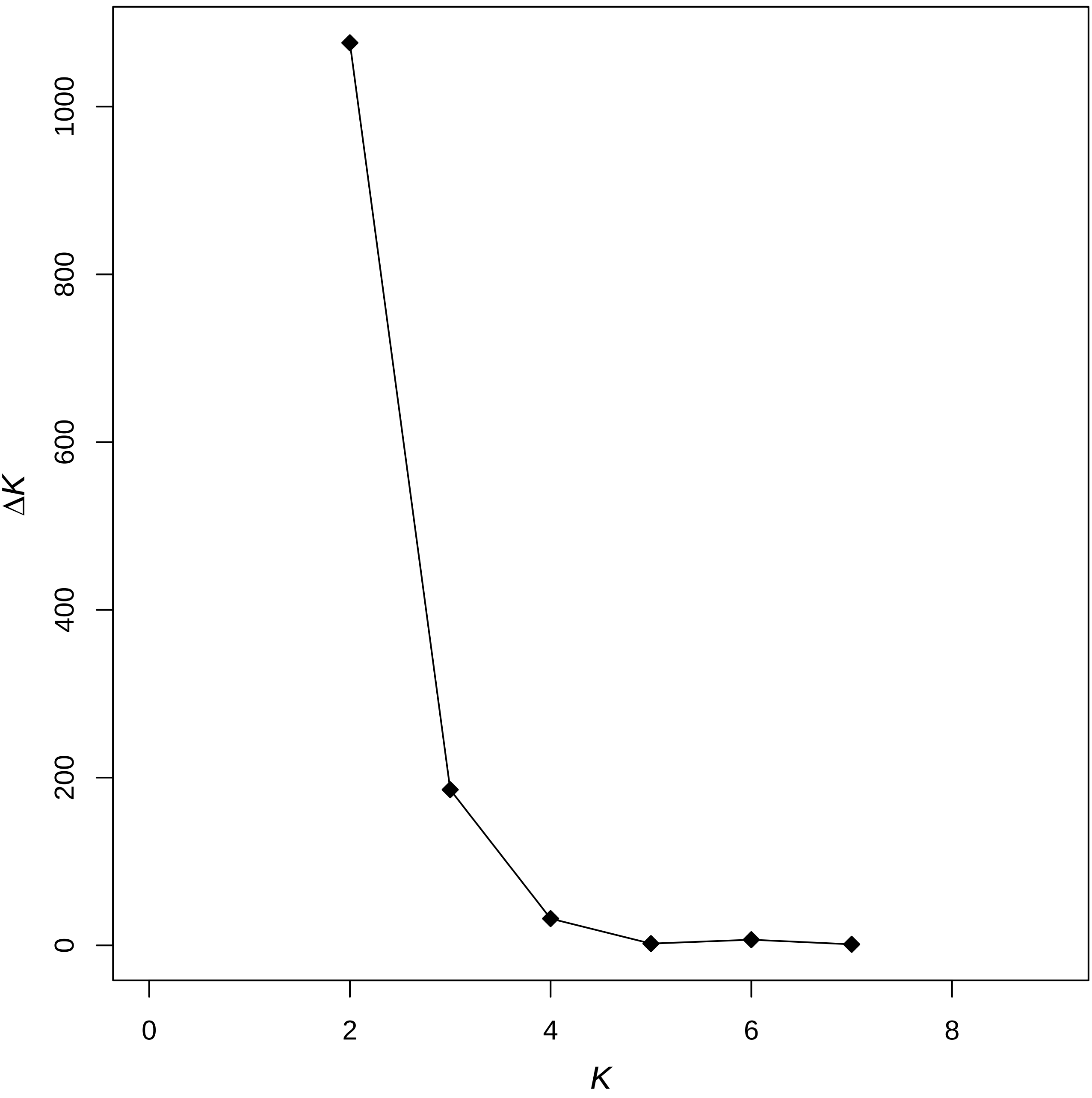

Fig. S6 ΔK calculated according to Evanno et al. (2005) for each K=1-8.

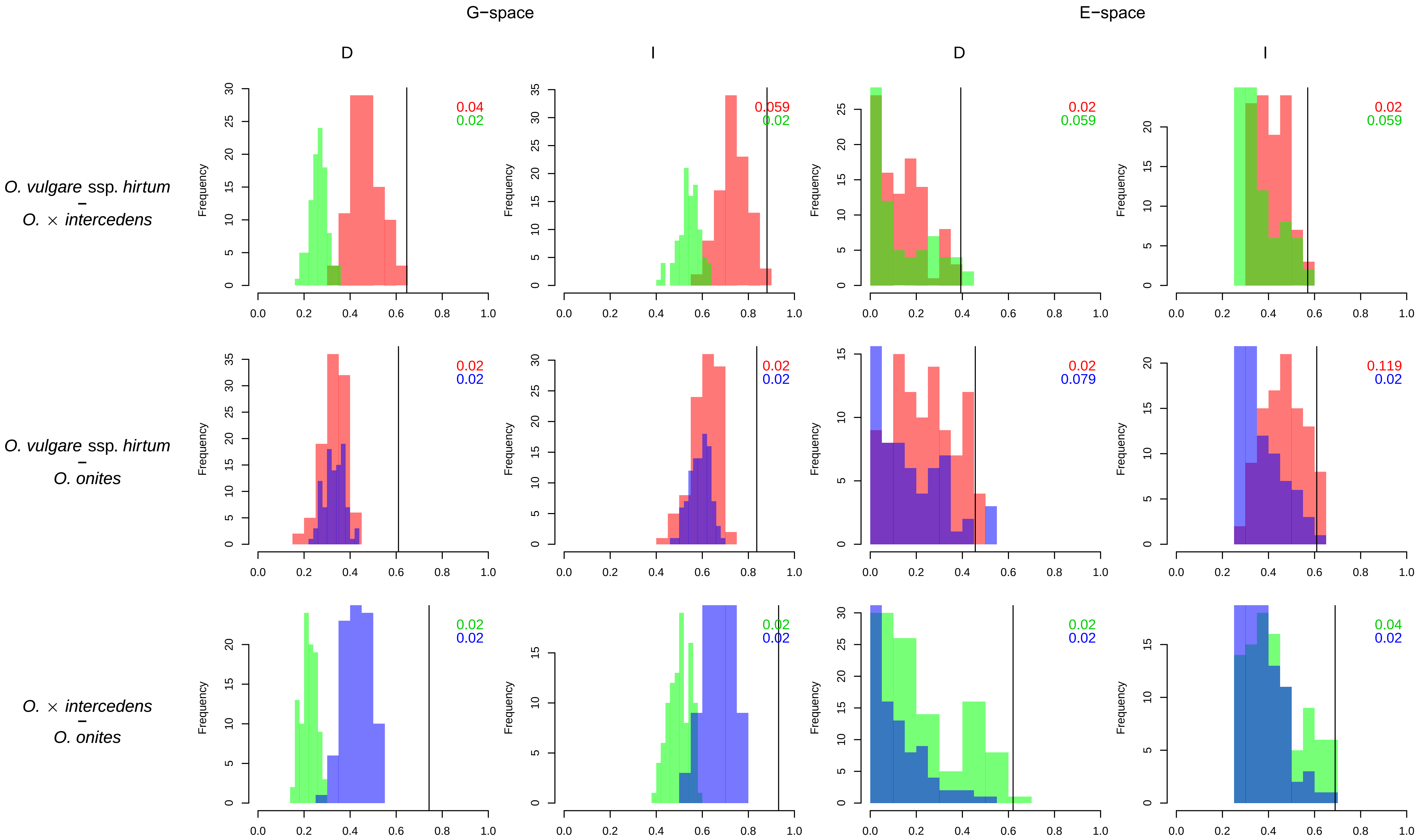

Fig. S7 Histograms of the simulated and observed D and I metrics for the G- and E-space similarity tests of M dataset. The different colors represent the focal taxon of each test, which was tested against the background of the second taxon: red, green and blue correspond to *O. vulgare* ssp. *hirtum*, *O.* × *intercedens* and *O. onites*, respectively. Vertical lines indicate the observed value of the measured metric. Numbers in the top right corner of each plot are the p-values of the respective tests.
