# Supplementary material for: Environmental (in)dependence of a hybrid zone: Insights from molecular markers and ecological niche modeling in a hybrid zone of Origanum (Lamiaceae) on the island of Crete
Source: Ecol Evol. 2016 Nov 16;6(24):8727–39. doi: 10.1002/ece3.2560 (PMC5192822; doi:10.1002/ece3.2560)
Supplement: Supplementary file 4 [file ECE3-6-8727-s004.pdf]

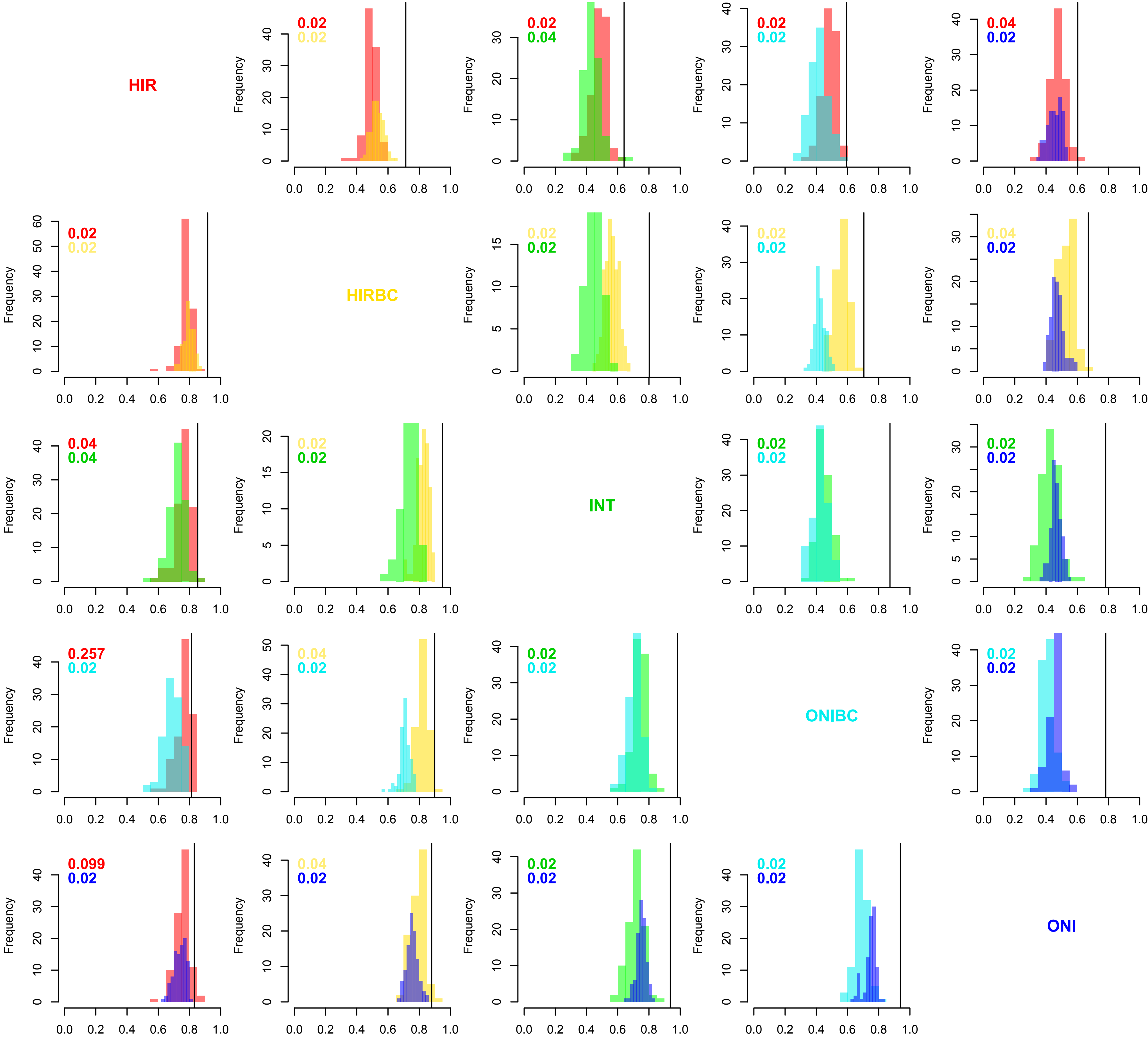

Fig. S8 Histograms of the simulated and observed D and I metrics for the G-space similarity tests of G data set. The different colors represent the focal entity of each test, which was tested against random points from the background of the second entity: red, yellow, green, cyan and blue correspond to group HIR, HIRBC, INT, ONIBC and ONI, respectively. Vertical lines indicate the observed value of the measured metric. Plots above the diagonal correspond to D metric, while those below the diagonal correspond to I. Numbers in the top right corner of each plot are the p-values of the respective tests. For details on group names, see main text.

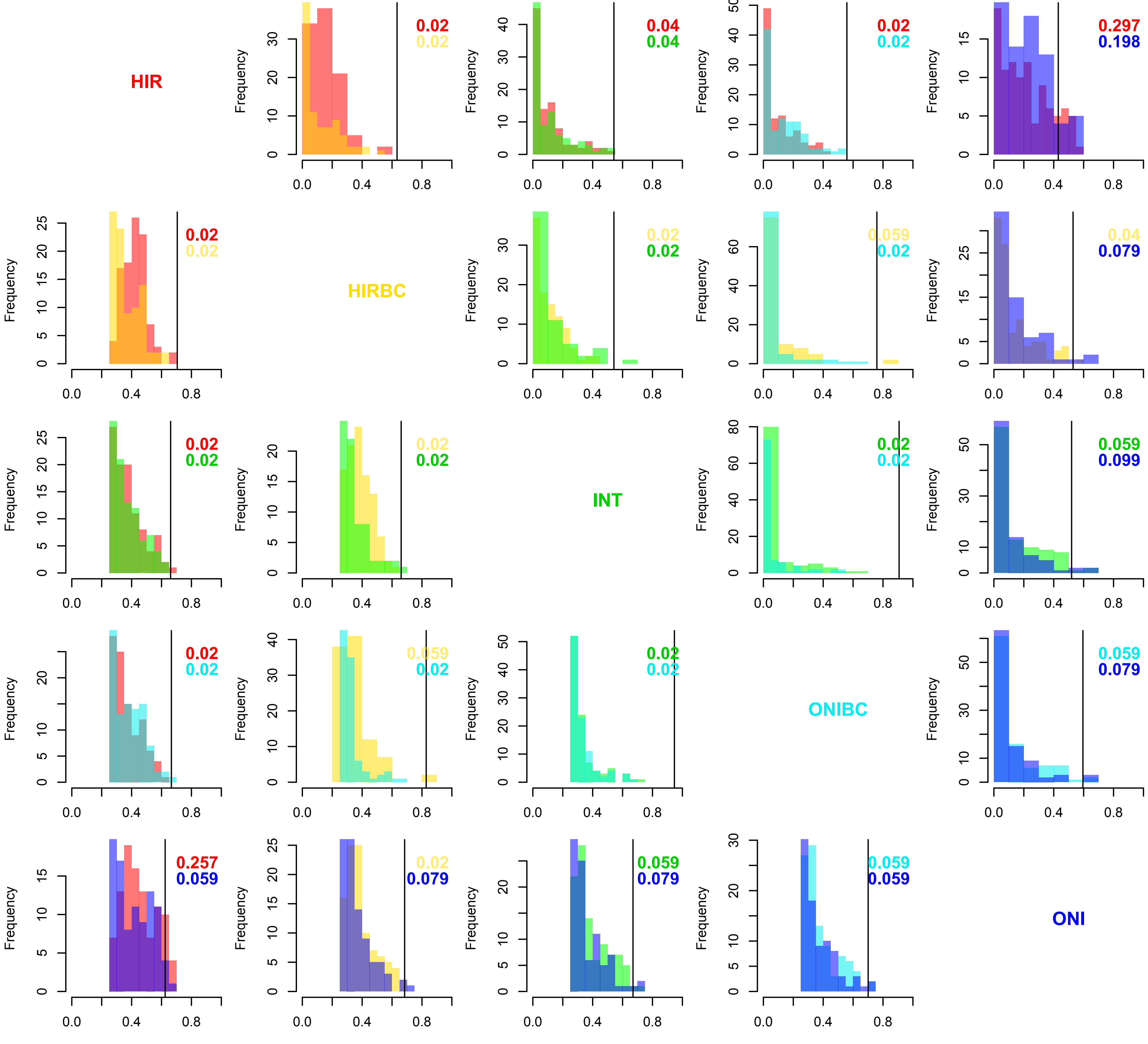

Fig. S9 Histograms of the simulated and observed D and I metrics for the E-space similarity tests of G data set. The different colors represent the focal entity of each test, which was tested against random points from the background of the second entity: red, yellow, green, cyan and blue correspond to HIR, HIRBC, INT, ONIBC and ONI, respectively. Vertical lines indicate the observed value of the measured metric. Plots above the diagonal correspond to D metric, while those below the diagonal correspond to I. Numbers in the top right corner of each plot are the p-values of the respective tests. For details on group names, see main text.
