# Supplementary material for: Environmental (in)dependence of a hybrid zone: Insights from molecular markers and ecological niche modeling in a hybrid zone of Origanum (Lamiaceae) on the island of Crete
Source: Ecol Evol. 2016 Nov 16;6(24):8727–39. doi: 10.1002/ece3.2560 (PMC5192822; doi:10.1002/ece3.2560)
Supplement: Supplementary file 5 [file ECE3-6-8727-s005.pdf]

Supplementary Material A. Here, we used 2 U of each *Eco*RI and *Mse*I restriction enzymes and 10 × reaction buffer NEB 4 (New England Biolabs, Hitchin, UK) to digest 100 ng of total DNA. The generated DNA fragments were ligated with *Eco*RI and *Mse*I adaptors using 2.5 U of T4 DNA ligase and 1 × T4 DNA ligase reaction buffer (New England Biolabs, Hitchin, UK). The resulting solution was diluted 5 times with TE buffer. Both pre-selective and selective amplifications were conducted in PTC 200 MJ Research thermocycler. In the pre-selective amplification we used 2.5 μL of digested-ligated DNA in a 22.5 μL mixture of 0.2 mmol/L dNTPs, 0.24 μmol/L of each *Eco*RI-A and *Mse*I-C primer, 1 U KapaTaq DNA polymerase, 5 × buffer (Kapa Biosystems, Wilmington, MA, USA) and ddH_2O following the PCR conditions described in Vos *et al*. (1995), modified by increasing the PCR cycles from 20 to 25 and adding a final extension step of 30 min at 72°C. The pre-amplified DNA product was diluted 10 times with TE buffer, and 2.5 μL of the diluted mixture were used as template for the selective amplification and added to a 7.5 μL mixture containing 0.2 mmol/L dNTPs, 0.04 μmol/L fluorescent *Eco*RI primer (*Eco*RI-ANN), 0.2 μmol/L *Mse*I primer (*Mse*I-CNN), 0.5 U Kapa Taq DNA polymerase, 5 × buffer (Kapa Biosystems, Wilmington, MA, USA) and ddH_2O, following the PCR conditions described in Vos *et al*. (1995).
