# Supplementary material for: Environmental (in)dependence of a hybrid zone: Insights from molecular markers and ecological niche modeling in a hybrid zone of Origanum (Lamiaceae) on the island of Crete
Source: Ecol Evol. 2016 Nov 16;6(24):8727–39. doi: 10.1002/ece3.2560 (PMC5192822; doi:10.1002/ece3.2560)
Supplement: Supplementary file 6 [file ECE3-6-8727-s006.docx]

Table S1 The number of collected individuals morphologically corresponding to *O.* × *intercedens*, *O*. *onites* and *O*. *vulgare* ssp. *hirtum* collected from the hybrid zone populations (A) and the allopatric populations (B).

(A)

| Taxa | Hybrid zone populations | | | | | | | | | | | | | | |
| --- | --- | --- | --- | --- | --- | --- | --- | --- | --- | --- | --- | --- | --- | --- | --- |
|  | ORI-1 | ORI-2 | ORI-3 | ORI-4 | ORI-5 | ORI-6 | ORI-7 | ORI-8 | ORI-9 | ORI-10 | ORI-11 | ORI-12 | ORI-13 | ORI-14 | ORI-15 |
| *O.* × *intercedens* | 4 | 6 | 6 | 6 | 5 | 6 | 6 | 3 | 3 | 6 | 5 | 2 | 3 | 6 | 6 |
| *O. onites* | 4 | 4 | 3 | 4 |  | 4 | 3 | 3 | 4 | 4 | 5 |  | 4 |  | 4 |
| *O. vulgare* ssp*. hirtum* | 4 |  |  | 4 | 4 |  | 2 | 2 | 4 | 4 | 4 | 5 | 4 | 4 | 4 |

(B)

| Taxa | Allopatric populations | | | | | | | | | | | |
| --- | --- | --- | --- | --- | --- | --- | --- | --- | --- | --- | --- | --- |
|  | ORI-16 | ORI-17 | ORI-18 | ORI-19 | ORI-20 | ORI-21 | ORI-22 | ORI-23 | ORI-24 | ORI-25 | ORI-26 | ORI-27 |
| *O.* × *intercedens* |  |  |  |  |  |  |  |  |  |  |  |  |
| *O. onites* | 4 | 4 | 4 | 4 | 4 | 3 |  |  |  |  |  |  |
| *O. vulgare* ssp*. hirtum* |  |  |  |  |  |  | 4 | 4 | 4 | 4 | 4 | 4 |

**Table S2** Key for the *Origanum* x *intercedens* calyx types. The different types (A-H) are shown in Fig. S3.

| Dichotomous key |  |
| --- | --- |
| 1. Calyx teeth not well developed | 2 |
| 1. Calyx teeth well developed | 4 |
|  |  |
| 1. Lower calyx teeth not well developed | Type G |
| 1. Upper calyx teeth not well developed | 3 |
|  |  |
| 1. One of the upper calyx teeth not well developed | Type H |
| 1. All upper calyx teeth not well developed | Type C |
| 1. Lower calyx teeth equaling, almost equaling or longer than the upper calyx teeth | 5 |
| 1. Lower calyx teeth clearly shorter than the upper calyx teeth | Type B |
|  |  |
| 1. Lower calyx teeth narrowly triangular | 6 |
| 1. Lower calyx teeth broadly triangular | 7 |
|  |  |
| 1. Lower calyx teeth almost reaching the margin of the upper calyx teeth | Type D |
| 1. Lower calyx teeth not reaching the margin of the upper calyx teeth | Type F |
|  |  |
| 1. Lower calyx teeth almost reaching the margin of the upper calyx teeth | Type E |
| 1. Lower calyx teeth not reaching the margin of the upper calyx teeth | Type A |

Table S3 The four primer pairs used in the AFLP technique, the fluorescent dyes of each *Eco*RI-ANN primer with their colors and the number of polymorphic markers per primer pair in the initial and final AFLP dataset.

| Code | Primer pair combinations | Fluorescent dyes (colors) | Polymorphic markers |
| --- | --- | --- | --- |
|  |  |  | Final data matrix (805 markers) |
| G | *Eco*RI-ACT / *Mse*I-CTT | HEX (green) | 210 |
| Y | *Eco*RI-AAG / *Mse*I-CAC | TAMRA (yellow) | 191 |
| B | *Eco*RI-AGG /MseI-CAA | FAM (blue) | 191 |
| R | *Eco*RI-AAG /*Mse*I-CTA | ROX (red) | 213 |

**Table S4** Environmental variables retained after screening of the initial 43 variables. This set was used for all subsequent analyses presented in this study.

| Variable name | Description |
| --- | --- |
| alt | altitude |
| bare | proportion of land covered by bare soil |
| east | aspect eastness |
| north | aspect northness |
| rmean | mean monthly rainfall |
| rmin | minimum monthly rainfall |
| s.IL | soil impermeable layer |
| s.SCC | subsoil calcium carbonate |
| s.SOC | subsoil organic carbon |
| s.TGYPS | topsoil gypsum |
| slope | slope |
| tmax | maximum temperature |
| trees | proportion of land covered by trees |

Table S5 Frequencies of species-specific markers in allopatric populations (A) and sympatric populations (B) of *O. οnites* and *O. vulgare* ssp*. hirtum*.

(A)

| Marker name | Allopatric  *O. οnites* | Allopatric  *O. vulgare* ssp*. hirtum* |
| --- | --- | --- |
| G34 | 0.87 | 0.04 |
| G43 | 1.00 | 0.04 |
| G44 | 0.91 | 0.04 |
| G51 | 1.00 | 0.08 |
| G73 | 0.13 | 0.92 |
| G143 | 0.83 | 0.00 |
| G207 | 0.83 | 0.00 |
| G215 | 0.96 | 0.00 |
| B27 | 0.87 | 0.04 |
| B28 | 0.04 | 0.88 |
| B54 | 0.96 | 0.08 |
| B75 | 0.87 | 0.04 |
| B164 | 0.83 | 0.00 |
| R82 | 1.00 | 0.08 |
| Y29 | 1.00 | 0.21 |

(B)

| Marker name | Sympatric  *O. οnites* | Sympatric  *O. vulgare* ssp*. hirtum* |
| --- | --- | --- |
| G51 | 0.98 | 0.07 |
| G215 | 0.85 | 0.07 |
| B27 | 0.83 | 0.07 |
| B39 | 0.80 | 0.04 |
| Β54 | 0.83 | 0.02 |
| R82 | 0.87 | 0.04 |

Table S6 Frequencies of species-specific markers in both allopatric and sympatric populations of *O. οnites* and *O. vulgare* ssp*. hirtum*.

| Marker name | Allopatric  *O. οnites* | Allopatric  *O. vulgare*ssp*. hirtum* | Sympatric  *O. οnites* | Sympatric  *O. vulgare*ssp*. hirtum* |
| --- | --- | --- | --- | --- |
| G51 | 1.00 | 0.08 | 0.98 | 0.07 |
| G215 | 0.96 | 0.00 | 0.85 | 0.07 |
| B27 | 0.87 | 0.04 | 0.83 | 0.07 |
| Β54 | 0.96 | 0.08 | 0.83 | 0.02 |
| R82 | 1.00 | 0.08 | 0.87 | 0.04 |

**Table S7** Model performance based on both 10-folding cross-validation and external evaluation using the separate dataset. The numbers indicate AUC ± standard deviation in the case of cross-validation and AUC in the case of external evaluation.

|  | Cross-validation | External evaluation |
| --- | --- | --- |
| Morphological Data |  |  |
| *O. vulgare* ssp. *hirtum* | 0.812 ± 0.111 | 0.864 |
| *O.* × *intercedens* |  |  |
| environmental variables only | 0.897 ± 0.064 | 0.926 |
| environmental variables and parental co-occurrence | 0.928 ±0.088 | 0.919 |
| *O. onites* | 0.829 ± 0.132 | 0.754 |
| Genetic Data |  |  |
| HIR (*q*₂ 0.9-1) | 0.812 ± 0.111 |  |
| HIRBC (*q*₂ 0.6-0.9) | 0.825 ± 0.142 |  |
| INT (*q*₂ 0.4-0.6) | 0.863 ± 0.068 |  |
| ONIBC (*q*₂ 0.1-0.4) | 0.887 ± 0.1 |  |
| ONI (*q*₂ 0-0.1) | 0.804 ± 0.124 |  |

**Table S8** Variable percent contribution and permutation importance for all environmental variables and parental occurrence variable, as given by Maxent.

| Variable code | Variable description | Percent contribution | Permutation importance |
| --- | --- | --- | --- |
| pocc | parental occurrence | 96.2 | 95.4 |
| rmin | minimum monthly rainfall | 1.1 | 2.1 |
| east | aspect eastness | 0.9 | 0.3 |
| slope | slope | 0.8 | 1.2 |
| north | aspect northness | 0.7 | 0.9 |
| trees | proportion of land covered by trees | 0.2 | 0.1 |
| rmean | mean monthly rainfall | 0.1 | 0.2 |
| s_TGYPS | topsoil gypsum | 0 | 0 |
| tmax | maximum temperature | 0 | 0 |
| bare | proportion of land covered by bare soil | 0 | 0 |
| alt | altitude | 0 | 0 |
| s_IL | soil impermeable layer | 0 | 0 |
| s_SCC | subsoil calcium carbonate | 0 | 0 |
| s_SOC | subsoil organic carbon | 0 | 0 |

**Table S9** Results of the Wilcoxon tests applied to each environmental variable for all possible pairs of the M and G data sets. The asterisks indicate significant divergence between the two entities under comparison in the respective variable. A minus sign (-) indicates that the entities were identical with respect to that particular variable and no test took place.

| Species pair | Environmental variable | | | | | | | | | | | | |
| --- | --- | --- | --- | --- | --- | --- | --- | --- | --- | --- | --- | --- | --- |
|  | alt | bare | east | north | rmean | rmin | s.IL | s.SCC | s.SOC | s.TGYPS | slope | tmax | trees |
| **M dataset** |  |  |  |  |  |  |  |  |  |  |  |  |  |
| *O. vulgare* ssp. *hirtum - O.* × *intercedens* | 0.232 | 0.708 | 0.772 | 0.942 | 0.942 | 0.385 | - | 0.096 | 0.288 | - | 0.800 | 0.169 | 0.490 |
| *O. vulgare* ssp. *hirtum - O. onites* | 0.318 | 0.832 | 0.715 | 0.366 | 0.438 | 0.051 | 0.317 | **0.028*** | 0.116 | 0.317 | 0.476 | **0.007*** | 0.116 |
| *O.* × *intercedens -O. onites* | 0.870 | 0.576 | 0.491 | 0.365 | 0.491 | 0.218 | 0.361 | 0.520 | 0.520 | 0.361 | 0.310 | 0.128 | 0.457 |
| **G dataset** |  |  |  |  |  |  |  |  |  |  |  |  |  |
| HIR - HIRBC | 0.203 | 0.891 | 0.746 | 0.746 | 0.351 | 0.556 | - | 0.378 | 0.737 | - | 0.482 | 0.556 | 0.469 |
| HIR - INT | 0.231 | 0.724 | 0.662 | 0.924 | 0.924 | 0.392 | - | 0.078 | 0.241 | - | 0.775 | 0.203 | 0.481 |
| HIR - ONIBC | 0.345 | 0.401 | 0.719 | 0.753 | 0.472 | 0.280 | - | **0.033*** | 0.111 | - | 0.589 | 0.053 | 0.620 |
| HIR - ONI | 0.319 | 0.833 | 0.716 | 0.367 | 0.438 | 0.052 | 0.317 | **0.028*** | 0.116 | 0.317 | 0.476 | **0.007*** | 0.116 |
| HIRBC - INT | 0.963 | 0.830 | 0.890 | 0.818 | 0.434 | 0.747 | - | 0.359 | 0.359 | - | 0.645 | 0.519 | 0.945 |
| HIRBC - ONIBC | 0.848 | 0.475 | 0.934 | 0.602 | 0.106 | 0.565 | - | 0.145 | 0.145 | - | 0.848 | 0.162 | 0.934 |
| HIRBC - ONI | 0.805 | 0.725 | 0.470 | 0.595 | 0.879 | 0.159 | 0.378 | 0.179 | 0.179 | 0.378 | 0.183 | **0.037*** | 0.371 |
| INT - ONIBC | 0.848 | 0.626 | 0.934 | 0.805 | 0.395 | 0.805 | - | 0.504 | 0.504 | - | 0.805 | 0.426 | 0.891 |
| INT - ONI | 0.834 | 0.584 | 0.403 | 0.447 | 0.543 | 0.254 | 0.378 | 0.646 | 0.646 | 0.378 | 0.323 | 0.119 | 0.529 |
| ONIBC - ONI | 1.000 | 0.364 | 0.514 | 0.380 | 0.144 | 0.380 | 0.434 | 0.812 | 0.812 | 0.434 | 0.251 | 0.458 | 0.444 |

Variable abbreviations: alt - altitude, bare - proportion of land covered by bare soil, east - aspect eastness, north - aspect northness, rmean - mean monthly rainfall, rmin - minimum monthly rainfall, s.IL - soil impermeable layer, s.SCC - subsoil calcium carbonate, s.SOC - subsoil organic carbon, s.TGYPS - topsoil gypsum, slope - slope, tmax - maximum temperature, trees - proportion of land covered by trees Genotypic groups (ranges of *q*_2_) : ONI 0-0.1, ONIBC 0.1-0.4, INT 0.4-0.6, HIRBC 0.6-0.9, HIR 0.9-1
