# Supplementary figures and images for: Environmental (in)dependence of a hybrid zone: Insights from molecular markers and ecological niche modeling in a hybrid zone of Origanum (Lamiaceae) on the island of Crete
Source: Ecol Evol. 2016 Nov 16;6(24):8727–39. doi: 10.1002/ece3.2560 (PMC5192822; doi:10.1002/ece3.2560)

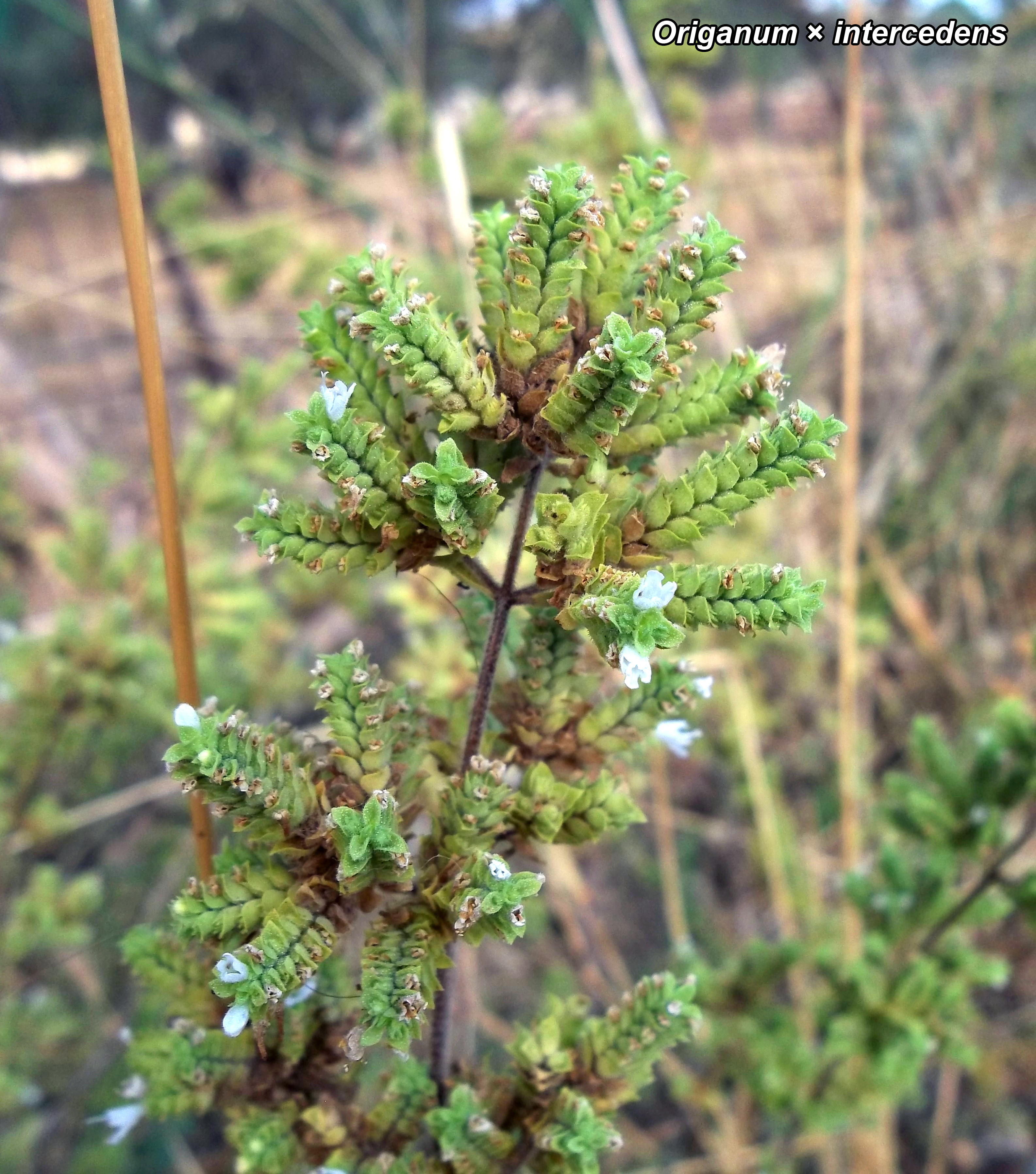

Supplement: Supplementary file 7 [file ECE3-6-8727-s007.tif]

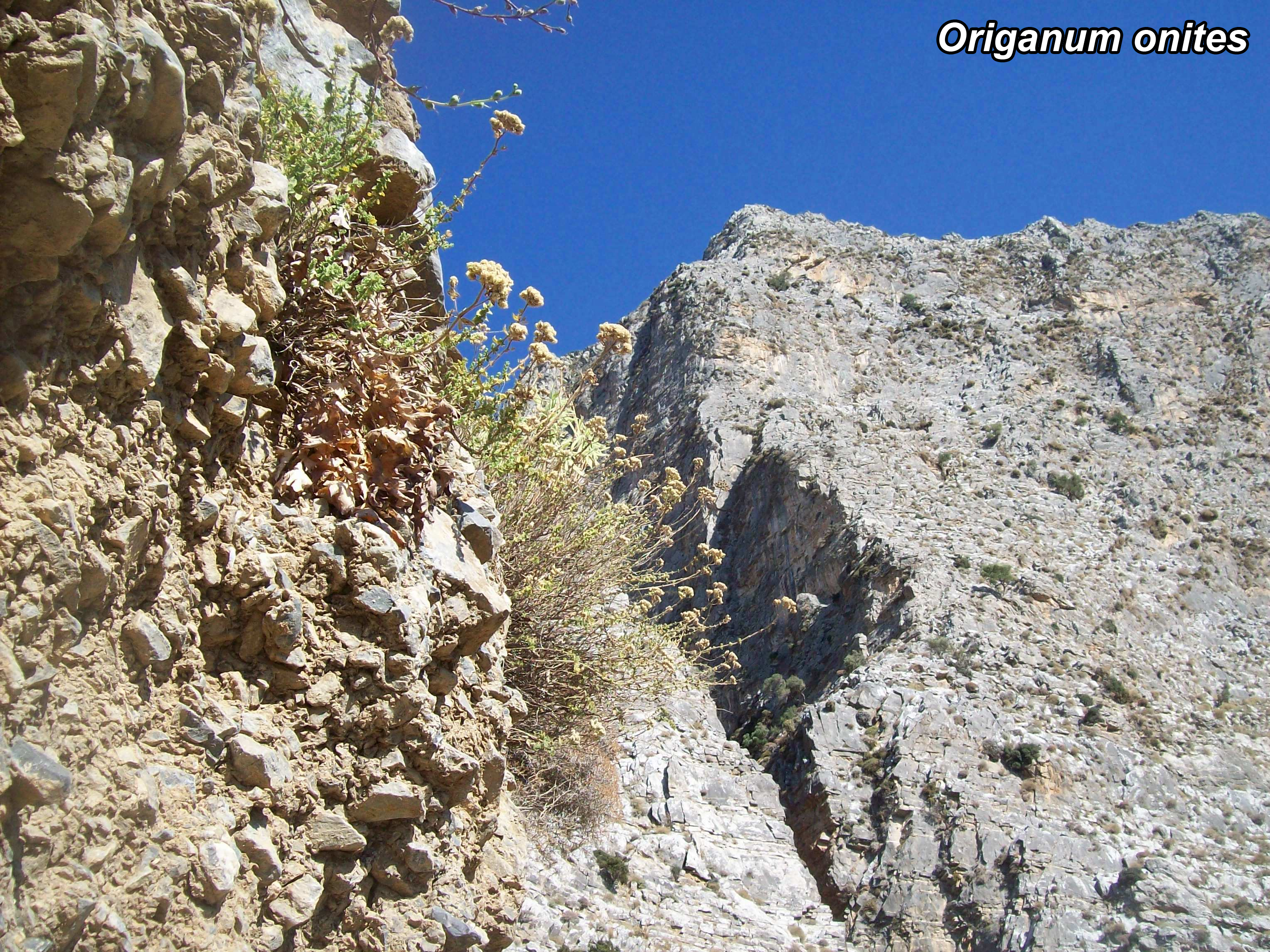

Supplement: Supplementary file 8 [file ECE3-6-8727-s008.tif]

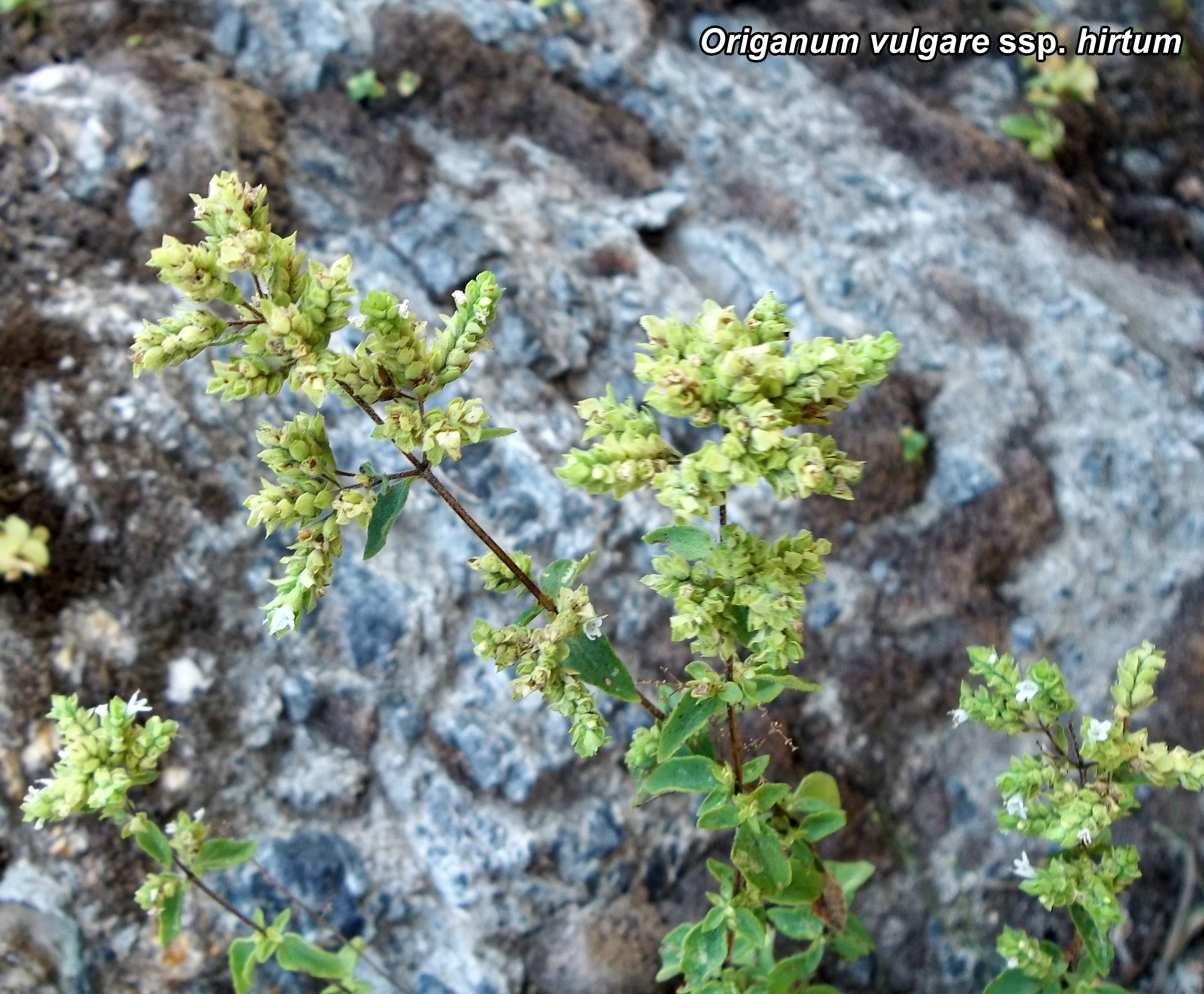

Supplement: Supplementary file 9 [file ECE3-6-8727-s009.tiff]
